# Systematic review of drug-drug interactions of delta-9-tetrahydrocannabinol, cannabidiol, and *Cannabis*

**DOI:** 10.3389/fphar.2024.1282831

**Published:** 2024-05-22

**Authors:** Rahul Nachnani, Amy Knehans, Jeffrey D. Neighbors, Paul T. Kocis, Tzuo Lee, Kayla Tegeler, Thomas Trite, Wesley M. Raup-Konsavage, Kent E. Vrana

**Affiliations:** ^1^ Department of Pharmacology, Penn State University College of Medicine, Hershey, PA, United States; ^2^ Department of Library, Penn State University College of Medicine, Hershey, PA, United States; ^3^ Department of Pharmacy, Penn State University College of Medicine, Hershey, PA, United States; ^4^ PA Options for Wellness, Harrisburg, PA, United States

**Keywords:** *Cannabis*, cannabinoids, thc, cbd, pharmacokinetics, narrow therapeutic index, drugdrug interactions

## Abstract

**Background:**

The recent exponential increase in legalized medical and recreational cannabis, development of medical cannabis programs, and production of unregulated over-the-counter products (e.g., cannabidiol (CBD) oil, and delta-8-tetrahydrocannabinol (delta-8-THC)), has the potential to create unintended health consequences. The major cannabinoids (delta-9-tetrahydrocannabinol and cannabidiol) are metabolized by the same cytochrome P450 (CYP) enzymes that metabolize most prescription medications and xenobiotics (CYP3A4, CYP2C9, CYP2C19). As a result, we predict that there will be instances of drug-drug interactions and the potential for adverse outcomes, especially for prescription medications with a narrow therapeutic index.

**Methods:**

We conducted a systematic review of all years to 2023 to identify real world reports of documented cannabinoid interactions with prescription medications. We limited our search to a set list of medications with predicted narrow therapeutic indices that may produce unintended adverse drug reactions (ADRs). Our team screened 4,600 reports and selected 151 full-text articles to assess for inclusion and exclusion criteria.

**Results:**

Our investigation revealed 31 reports for which cannabinoids altered pharmacokinetics and/or produced adverse events. These reports involved 16 different Narrow Therapeutic Index (NTI) medications, under six drug classes, 889 individual subjects and 603 cannabis/cannabinoid users. Interactions between cannabis/cannabinoids and warfarin, valproate, tacrolimus, and sirolimus were the most widely reported and may pose the greatest risk to patients. Common ADRs included bleeding risk, altered mental status, difficulty inducing anesthesia, and gastrointestinal distress. Additionally, we identified 18 instances (58%) in which clinicians uncovered an unexpected serum level of the prescribed drug. The quality of pharmacokinetic evidence for each report was assessed using an internally developed ten-point scale.

**Conclusion:**

Drug-drug interactions with cannabinoids are likely amongst prescription medications that use common CYP450 systems. Our findings highlight the need for healthcare providers and patients/care-givers to openly communicate about cannabis/cannabinoid use to prevent unintended adverse events. To that end, we have developed a free online tool (www.CANN-DIR.psu.edu) to help identify potential cannabinoid drug-drug interactions with prescription medications.

## Introduction

Several cannabis-based medications have received U.S. Food and Drug Administration (FDA) approval for use in patients, including dronabinol (Marinol^®^), cannabidiol (Epidiolex^®^), nabilone (Cesamet^®^), and the investigational drug nabiximols (Sativex^®^). Moreover, local/state jurisdictions have approved medicinal and recreational cannabis and cannabis extracts and recent years have seen increased use of cannabidiol (CBD) oil as an over-the-counter dietary supplement. With this expanding use of cannabis and cannabinoids, clinicians, researchers, and patients require a better understanding of the potential for cannabinoids to interact with other medications through drug-drug interactions to produce unintended side effects and/or adverse drug reactions (ADRs).

The legal ramifications and public stigma associated with use of cannabis have resulted in a lack of rigorous, well-controlled, research studies on the medicinal properties of cannabis. Most of the data collected on the medical use of cannabis remain anecdotal, individual case studies, and small clinical trials. Furthermore, due to stigma surrounding cannabis use, patients are at times reluctant to inform their physicians of their recreational cannabis use. While the major cannabinoid components of cannabis (delta-9- tetrahydrocannabinol (THC) and CBD) are generally viewed by the public as non-toxic, there is the potential for these compounds to interact with and alter the pharmacokinetics of other medications (Kocis and Vrana, 2020; Lopera et al., 2022).

Previously, we and others have reported potential drug-drug interactions between cannabis and prescription medications, based upon enzyme metabolism (Kocis and Vrana, 2020; Lopera et al., 2022). The hepatic enzymes typically employed to metabolize xenobiotics such as components of *Cannabis*, cannabinoids, and many prescribed medications are the phase I cytochrome P450 (CYP450) enzymes CYP3A4, CYP2C9, and CYP2C19 (Kocis and Vrana, 2020). Other minor CYP enzymes are also involved in metabolizing cannabinoids to a lesser extent. This systematic review focused on these enzymes as well as the phase II UGT conjugation enzymes, which have been reported to interact with cannabidiol (Nasrin et al., 2021). When the hepatic system is faced with simultaneously metabolizing several substrates with these enzymes, unexpected pharmacokinetic effects and downstream physiological events may occur. In fact, any insult or pharmacogenetic polymorphism may create unintended consequences in drug action. This risk is demonstrated by previous *in vitro* (Chen et al., 2000; Honda et al., 2011) reports of CYP-driven metabolism of key biological building blocks. Additionally, CYP metabolism alterations are implicated in human toxicology as a potential for unforeseen medication effects, as demonstrated by post-mortem analyses of victims of overdose and suicide where differential genotypes of these metabolizing enzymes were associated with altered patterns of use, harm, and death (Koren et al., 2006; Vevelstad et al., 2016; Rahikainen et al., 2018; Di Nunno et al., 2021). Risk of harm is especially higher when considering the potential for narrow therapeutic index (NTI) medications, which are those with higher potential for adverse events when improperly dosed or when blood levels are unexpectedly altered. For this reason, a systematic review considering a variety of NTI drugs interacting with cannabinoids was conducted to understand the potential dose alterations and adverse events associated with concomitant use. Here, we identified 31 reports where cannabis or cannabinoid use alters the pharmacokinetics of prescription medications and/or produces ADRs.

## Methods

### Database search strategy

This systematic review was performed according to standards as described in the Preferred Reporting Items for Systematic Reviews and Meta-Analyses (PRISMA) guidelines (Moher et al., 2009). The aim of this systematic review is to evaluate the existing literature on drug-drug interactions (DDIs) of 57 identified medications (Kocis and Vrana, 2020) with cannabis and cannabinoids. Search terms were chosen based on the medications listed in Table 1. Additional terms were harvested by searching for the drug names in Micromedex-IBM^®^, the National Libraries of Medicine (NLM) Drug Information Portal and by examining search strategies published in the Cochrane Library of Systematic Reviews on similar topics. Several databases (MEDLINE, Embase, and Cochrane Central Register of Controlled Trials) were searched with the assistance of a medical librarian to identify articles published in all years since the beginning of time until 23 February 2023. The following keywords were used: cannabis, cannabidiol, marijuana, and drug interaction as well as the medications listed in Table 1. Furthermore, reference lists of relevant articles were searched manually for additional studies. Complete searches from MEDLINE are provided in Supplementary Appendix S1. No protocol exists for this systematic review. The completed PRISMA checklist for this systematic review is available in Supplementary Appendix S3.

**TABLE 1:** List of 57 Narrow Therapeutic Index Medications from Kocis and Vrana (2020). Of this list, bold compounds are those that were identified by our systematic review as having a documented interaction.

Acenocoumarol	Clomipramine	Diphenadione	**Fentanyl**	**Nortriptyline**	Temsirolimus
Alfentanil	**clonidine**	dofetilide	fluindione (VKA)	paclitaxel	**theophylline**
aminophylline	clorindione (VKA)	dosulepin	fosphenytoin	**phenobarbital**	thiopental
amiodarone	cyclobenzaprine	doxepin	**imipramine**	phenprocoumon	tianeptine
**amitriptyline**	cyclosporine	ergotamine	**levothyroxine**	phenytoin	trimipramine
amphotericin B	dabigatran etexilate	esketamine	lofepramine	pimozide	**valproic acid**
argatroban	**desipramine**	ethinyl estradiol (oral contraceptives)	melitracen	**propofol**	**warfarin (VKA)**
Busulfan	dicoumarol	ethosuximide	meperidine	quinidine	
**carbamazepine**	digitoxin	ethyl biscoumacetate	mephenytoin	**sirolimus**	
Clindamycin	dihydroergotamine	**everolimus**	mycophenolic acid	**tacrolimus**	

### Study selection

Authors (in pairs) independently screened titles and abstracts and selected articles for inclusion through full-text evaluation. Any unresolved inconsistency was resolved by a third reviewer. Drug-drug interactions were defined as interactions between cannabis that resulted in altered pharmacokinetics for prescription medications or appearance of ADRs.

### Eligibility criteria

Inclusion criteria were: English language and reported adverse event, altered pharmacokinetics, or adjustment in care when prescription drugs were co-administered with a cannabis product or cannabinoid. Exclusion criteria were: non-English language, no cannabis product mentioned, and no documented interaction with NTI prescription medication. Conference abstracts, presentations, unpublished studies, animal studies and studies whose full text was not able to be obtained were also excluded. Initial screening of identified abstracts was conducted by two independent investigators, followed by a full-text review if either reviewer deemed that a citation met inclusion criteria. All included studies were agreed upon by both reviewers. Once relevant citations were identified, a team member (not associated with the original selection) abstracted the relevant information and summarized the findings for a class of medications. The entire team edited the final document.

### Data extraction

A data extraction form was compiled for all studies to be included. Authors extracted data independently, including sample demographic information, drug information, cannabis use, pharmacological parameters, and existence of adverse events. When a study did not report the sex demographics of their study population, we did not include those values into our subdivided analysis, but included the participants in our total participant count. The extracted information is summarized in Table 2. Because the studies reviewed are highly heterogenous in design and reported outcomes, formal meta-analysis was not appropriate.

**TABLE 2:** Study characteristics of thirty-one reviewed articles describing drug-drug interactions with prescribed medications and cannabinoids.

Section	References	Study type	Subject sample (n)	Cannabis user (n)	Male cannabis users (n)	Female cannabis users (n)	Age range	ADRs (n)	ADR descriptions	Medication	Data Collection	Cannabis/Cannabinoid type, form of ingestion, frequency	Cannabis/Cannabinoid dose	Change in medication levels?
Xanthine Derivatives	Jusko	Prospective	57	14	8	6	19 to 27	0	NA	aminophylline (theophylline)	serum and saliva collection over time	Smoking >2x/week for several months	Not reported (N.R.)	Decrease in half-life; Increase in clearance
Anesthetics	Karam	Case Report	1	1	1	0	35	0	NA	morphine, paracetemol, ketorolac	Patient interview	Smoking >3x/week for 20y	NR	Doubled anesthetic requirements
	Symons	Case Report	1	1	1	0	34	1	Convulsion during induction and emergence from anesthesia	fentanyl, propofol, midazolam, ketorolac	Patient interview	Smoked cannabis night before surgery	NR	Increased anesthetic requirements
	Gregg	Cross-over	10	5	*	*	21 to 30	5	Smokers of cannabis had sustained tachycardia post-anesthesia vs. non-smokers	Atropine, fentanyl, diazepam, N_2_O gas, methohexital, lidocaine, epinephrine	Patient interview	Smoked cannabis within 72 h of operation	NR	None
	Flisberg	Prospective	60	30	30	0	18 to 50	0	NA	Propofol	Patient interview, Bispectral index, insertion of laryngeal mask	Smoking >1x/week for >6mo	NR	Increased propofol requirements to insert laryngeal mask
	Manini	Cross-over	17	12	6	6	40 to 49	0	NA	Fentanyl	Serum and urinary monitoring of: fentanyl, CBD, cortisol	CBD oil, oral, dosed once	0, 400mg, or 800 mg CBD	No interactions reported between CBD and fentanyl
	Imasogie	Case-Control	318	151	105	46	18 to 71	NR	NA	propofol	patient interview, chart review	any cannabis, frequency ranged from occasional to daily	NR	Cannabis users required on average 40% more propofol
	King	Retrospective	46	23	5	18	41.1 [mean]	NR		Propofol, ketamine, fentanyl, glycopyrrolate, benzocaine, lidocaine	chart review	any cannabis, varying frequency reports	NR	no significant difference in any anesthetics
Anticoagulants	Cortopassi	Case Report	1	1	1	0	46	0	NA	warfarin	INR, monitoring of warfarin and CBD dose	CBD (Epidiolex^®^)	20 mg/kg/day	20% dose reduction of warfarin
	Yamreudeewong	Case Report	1	1	1	0	56	1	Upper gastrointestinal bleed, nosebleed, easy bruising, syncope	warfarin	INR, monitoring warfarin dose	smoking 3–4x/week x 4 weeks	NR	frequent dose adjustments and hospitalizations
	Damkier	Case Report	1	1	1	0	27	0	NA	warfarin	INR, monitoring warfarin dose	frequent cannabis smoking	NR	no adjustments needed
	Hsu	Case Report	1	1	1	0	35	0	NA	warfarin	INR	edibles, smoking x 1 month	NR	acute adjustments needed
	Grayson	Case Report	1	1	1	0	44	0	NA	warfarin	INR, monitoring of warfarin and CBD dose	CBD (Epidiolex^®^)	5 mg/kg/day and doubled every 2 weeks	30% dose reduction of warfarin
	Brown	Case Report	1	1	1	0	67	1	milld dry mouth and transient dizziness	warfarin, nortriptyline, and others	patient history, INR self testing	sublingual CBD oil (5mg/1 mL) and sublingual 50:1 THC:CBD oil, 4.9 mg THC and 0.1 mg CBD/mL; several times daily under tongue	7.35 mg THC/day 10.15 mg CBD/day escalated to 14.7 mg THC and 10.3 mg CBD per day	27% dose reduction needed for warfarin
	Thomas	Case Report	1	1	1	0	85	0	NA	warfarin	INR, serum levels of THC, CBD	THC and CBD, oromucosal oil, daily and as needed	0.3 mg THC/5.3 mg CBD daily with 0.625 mg THC/0.625 mg CBD as needed	None needed
Antidepressants	Wilens	Case Series	4	4	4	0	15 to 18	4	altered mental status, hallucinations, depersonalization, dry mouth, racing heart, shortness of breath	nortriptyline, desipramine, clonidine	Patient interview	marijuana cigarettes	1–2 marijuana cigarettes, varied	NR
	Kizer	Case Report	1	1	1	0	26	1	disorientation, anxious, dizziness, tachycardia	imipramine	Patient interview	marijuana cigarette	1 marijuana cigarette	NA
Transplants	Ebrahimi-Fakhari	Retrospective	25	25	18	7	3 to 43	10	diarrhea, drowsiness, severe mouth sores, acne, ankle swelling, sinusitis, abdominal pain, elevated transaminases, and increased phenytoin level	everolimus (18 patients) and sirolimus (7 patients), phenytoin	chart review	cannabidiol oral solution	cannabidiol 5–20 mg/kg/day	everolimus and sirolimus levels were higher in 76% of patients after cannabidiol treatment
	Hauser	Case Report	1	1	1	0	67	1	diarrhea, body stiffness, tremors, and altered mental status, required ICU transfer	tacrolimus	chart review	marijuana gummies	NA	tacrolimus level higher than expected
	Moadel	Case Report	1	1	0	1	48	1	Encephalopathy (agitation and delirium) secondary to tracrolimus toxicity	tacrolimus	chart review	Taking 2–4 medical marijuana lozenges per day up to the time of transplant. Denied any lozenges during hospital admission	1 lozenge contains 10 mg THC and 1 mg CBD	tacrolimus level ranged from 50%–200% of ideal dose
	Leino	Case Report	1	1	0	1	32	1	tacrolimus toxicity (as defined as elevated serum creatinine)	tacrolimus	case report; open label study	cannabidiol oral solution	2000–2,900 mg/day cannabidiol	Higher than normal creatinine, required dose adjustment of CBD
	Cuñetti	Case Series	7	7	4	3	58 to 75	3	nausea, dry mouth, dizziness, drowsiness, and intermittent episodes of ‘heat’ (warmth)	tacrolimus	lab chart review and pain index scores	cannabidiol oral solution	Initial dose of cannabidiol 100 mg/day with progressive increase up to 300 mg/day	NA
Anticonvulsants	Klotz	Case Series	5	5	4	1	10 to 54	0	NA	various AEDs, including valproate, phenobarbital	PK parameters assessed of NTI drugs	CBD	started 5 mg/kg/day and increased to 25–50 mg/kg/day	NR for NTI drugs
	Wiemer-Kruel	Case Report	1	1	0	1	6	0	NA	Everolimus	trough everolimus levels by serum collection	CBD	200 mg/day up to 500 mg/day	increased and unstable everolimus levels with CBD
	Devinsky	RCT	34	27	11	16	4 to 11	20	Some liver enzyme elevation with CBD and valproate, CBD alone pyrexia, somnolence, sedation, ataxia, vomiting	Valproate and other AEDs	Serial serum collection and analysis for drug exposure	CBD	5, 10, 20 mg/kg/day bid	NA
	Ben-Menachem	RCT	34	28	17	11	17 to 54	22	most common diarrhea, mild	stiripentol or valproate	Serial serum collection and PK analysis	CBD (Epidiolex^®^)	20 mg/kg/day for 26 days	decrease of AUC and Cmax of valproate (17% and 13%)
	Morrison	RCT	77	77	50	27	26 to 35	12	rash including severe rash, menstrual discomfort, drunk feeling	Valproate and other AEDs	Serial serum collection and PK analysis	CBD (Epidiolex^®^)	750 mg bid for CBD	No effect of CBD on valproate
	Gaston	Prospective Trial	81	81	41	40	2 to 62	0	NA	19, included valproate	baseline serum collections and then at each visit for antiepileptic drugs	CBD (Epidiolex^®^)	initiated at 5 mg/kg/day and increased every 2 weeks to 50 mg/kg/day	Valproate not recorded
	McNamara	Retrospective	87	87	44	43	1.2 to 19.8	9	elevated liver enzymes, thrombocytopenia, easy bruising, gum bleeding, hematuria	Valproate and other AEDs	Serial serum collection	CBD (Epidiolex^®^)	Highest dose CBD 13.6 ± 5.0 mg/kg/day	Required reduction of either valproic acid or CBD, one patient had to stop CBD completely
	Caceres Guido	Clinical Trial, Phase 1	12	12	2	10	2.5 to 17.2	NR	NR	Valproate, levothyroxine, and other AEDs	serum CBD levels	CBD (Epidiolex^®^), po or ng tube, 2x/day	initiated at 2 mg/kg/day and increased every 3 weeks	No reported changes
	Ridout	Case Report	1	1	1	0	37	0	0	carbamazepine, olanzapine, temazepam	patient interviews, carbamazepine serum levels	smoking 1–2 joints daily	none reported	20% increase and decrease depending on cannabis use
		Totals	889	603	361	237	2 to 85	92						
Total Study Number	31													

**Legend and Abbreviations**: RCT, randomized controlled trial; NR, not reported; NA, not applicable; CBD, cannabidiol; ICU, intensive care unit; AED, anti-epileptic drug; INR, international normalized ratio; THC, delta-9-tetrahydrocannabinol.

### Quality of evidence assessment

To rank quality of pharmacokinetic evidence, we utilized a descriptive approach (Good, Fair, Poor) for each study, adapted from the US Preventative Service Task Force (USPSTF) (U.S. Preventive Services Task Force, 2018). We developed a 10-point scale and assigned numerical scores to six categories necessary for informed clinical decision-making regarding drug-drug interactions for each study in the review. These categories include: sample size, reporting drug pharmacokinetics, reporting cannabinoid pharmacokinetics, reporting drug and cannabinoid dosing and frequency, balanced male and female cohorts in samples (defined as including 40%–60% female subjects), and control subjects. Studies received summary grades determined by presence or absence of these metrics: Good (8–10), Fair (4–7), and Poor (0–3). Our search yielded many case reports and case series, which were analyzed using a modified version of the protocol of toxicology case reports (Nambiema et al., 2021). In accordance with these guidelines, all case reports/series receive the lowest rating of evidence in comparison to other studies; however, the inclusion and exclusion of important pharmacokinetic metrics are still rated for the case reports and series to extract thorough pharmacologic data. The quality of each study was assigned independently by two authors and any differences were resolved through discussion with a third author. The detailed grading, rating, and category description for each study can be found in Supplementary Appendix S2.

## Results

### Screening results

**FIGURE 1:**
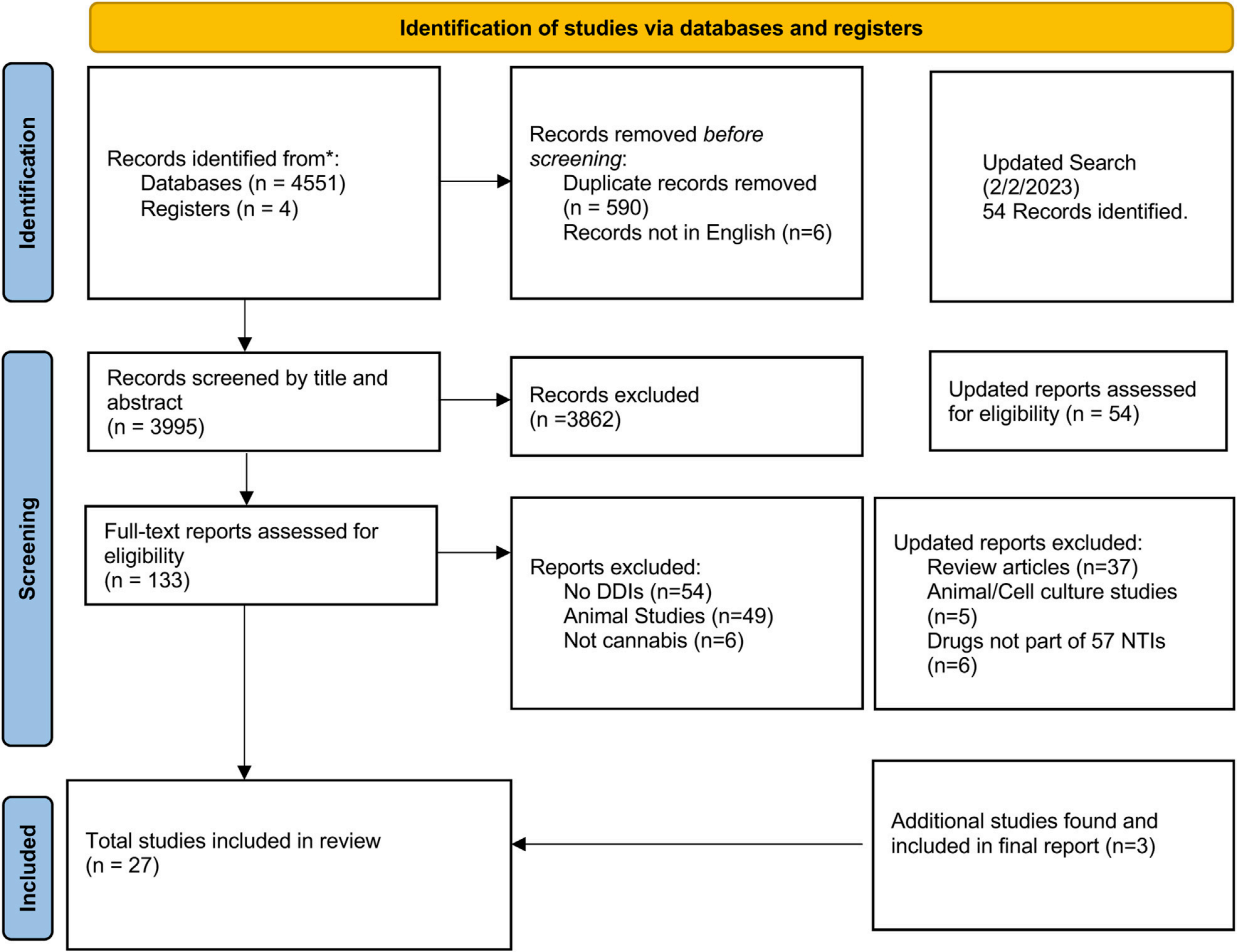
Search and selection results of studies reporting *Cannabis* and cannabinoid interactions with predicted narrow therapeutic index prescription medications using PRISMA 2020 Guidelines.

As shown in Figure 1, our initial search strategy identified 4,600 reports. Six reports were not included due to non-English language. After removing duplicates and screening by title and abstract, we assessed 151 full-text reports for eligibility. From these, 31 reports met inclusion and exclusion criteria–representing 889 subjects and 603 cannabis or cannabinoid users.

### Demographic results

The sex demographics of these samples are 59.9% male (*n* = 361/603) and 39.3% female (*n* = 237/603). One study did not report sex demographics in their sample (*n* = 5/603). Few studies reported on race and ethnicity demographics. There was a wide age range in the studies selected: ages 1.2 to 85. In total, there were 92 adverse drug reactions (ADRs) in the cannabis/cannabinoid user groups across all studies which are detailed in Table 2. The majority (18/31, 58%) of reports were case reports or case series, and the remaining 13 reports were comprised of safety trials and retrospective chart reviews.

### Results by drug class

To stratify the results of our search, we subdivided the 57 narrow therapeutic index (NTI) medications into drug class and/or indication. Sixteen of 57 NTI medications were reported in the included manuscripts and make up the following sections: methylxanthine derivatives, anesthetics and analgesics, anticoagulants, antidepressants, transplant medications, and anticonvulsants.

### Methylxanthine derivatives

Theophylline and methylxanthine derivatives are noteworthy for common drug-drug interactions. One pharmacokinetic study (Jusko et al., 1978) reported changes to theophylline half-life and clearance in three groups: cannabis non-tobacco smokers (*n* = 7), cannabis and cigarette smokers (*n* = 7), and control subjects (*n* = 43). The half-life of theophylline was 8.1 h in the control group, a significantly lower 5.9 h in the cannabis group (*p* < 0.05) and a comparable 5.7 h in the tobacco smoker group (*p* < 0.01). The mean clearance of theophylline increased from 51.8 mL/kg/hr (SD = 20.8) in nonsmokers to 73.3 mL/kg/hr (SD = 30.7) in cannabis users. Dual users experienced a clearance of 92.7 mL/kg/hr (SD = 25.3) (*p* < .05).

### Anesthetics and analgesics

We identified seven studies (prospective, retrospective, case reports) documenting clinical examples of cannabinoid interactions with propofol and fentanyl. Flisberg and colleagues led one prospective, randomized, single-blind trial of 60 male patients divided into two groups, cannabis users (*n* = 30) and non-cannabis users (*n* = 30), and assessed the requirements for propofol to induce anesthesia and insert a laryngeal mask (Flisberg et al., 2009). While there was no significant difference in propofol dose required to achieve induction between the two groups, cannabis users required significantly higher doses of propofol to insert the laryngeal mask (*p* < 0.04). Imasogie and colleagues conducted a case-control study of endoscopy patients with or without historic cannabis exposure (Imasogie et al., 2021). The researchers studied the propofol dose necessary to induce anesthesia in patients with varying self-reported cannabis frequency: none (controls), occasional, monthly, weekly, and daily. There was a dose-dependent significant association with dose necessary to induce anesthesia and cannabis frequency (*p* < 0.01). Daily cannabis users required an estimated 75% increase in propofol dose by weight. Contrary to these two trials, King and colleagues conducted a retrospective review of anesthetic requirements of patients undergoing esophagogastroduodenoscopy and self-reported cannabis use (King et al., 2021). The researchers studied the required doses of propofol, fentanyl, ketamine, and other anesthetics and, in this case, reported no significant difference between groups for required dose of anesthetic nor differences in post-procedure complications. Finally, two case reports describe increased dose requirements for anesthesia in cannabis users. Karam and colleagues (Karam et al., 2015) found increased propofol induction requirements and morphine maintenance requirements in a 35-year-old male chronic cannabis user. Symons (2002) reported a 34-year-old man who required three extra boluses of propofol, higher than usual concentrations of sevoflurane, and suffered short convulsions during induction and recovery. Post-operatively, this patient admitted to smoking cannabis the night before his surgery.

Manini and colleagues conducted a double-blind placebo-controlled trial (Manini et al., 2015) to determine the safety of fentanyl co-administration with CBD. Two doses of CBD pretreatment (400 and 800 mg) and two doses of fentanyl (0.5 mg/kg and 1.0 mg/kg) were tested in 17 (9 male, 8 female) subjects. The authors found no differences in most CBD pharmacokinetics, but urinary clearance of CBD was significantly reduced when co-administered with 1.0 mg/kg fentanyl (*p* = 0.02).

Gregg and colleagues published two studies on the effects of THC related to oral surgery (Gregg et al., 1976). They reported vital signs in the perioperative period in five cannabis smokers (within 72 h of surgery) and five nonsmokers after induction and maintenance of anesthesia using propofol, diazepam, methohexital, and nitrous gas. The researchers found no differences in blood pressure or blood gas readings; however, cannabis smokers had significantly increased (*p* < 0.05) peak postanesthetic heart rate (136.8 bpm) compared to nonsmokers (104.6 bpm).

### Anticoagulants

Seven case reports identified interactions between cannabinoids and warfarin; six out of seven subjects required warfarin dose adjustments and two experienced adverse effects from concomitant cannabis use. Of these cases, cannabis was responsible for interactions in 5 cases (2 smoking, 2 oral, and 1 sublingual) and CBD was involved in the other 2 reports (1 Epidiolex^®^ and 1 commercial CBD oil). The International Normalization Ratio (INR) is used as one measure of warfarin efficacy, and patients taking the medication must maintain a discrete and stable INR to prevent adverse events, the most dangerous being nervous system bleeds (hemorrhagic stroke and spinal cord bleed).

Yamreudeewong and colleagues (Yamreudeewong et al., 2009) report a 56-year-old male taking warfarin with a stable INR who started “smoking more marijuana than usual to self-treat depressive symptoms.” He experienced upper gastrointestinal bleeding, nosebleed, and increased bruising during the period of increased consumption. His INR values were found to be supratherapeutic at 9.7–11.6. Clinicians withheld warfarin to stabilize values and during 9 months of cannabis cessation, the subject had stable INR values between 1–4. Cortopassi (2020) reported a 46-year-old male taking warfarin who started Epidiolex^®^ (CBD) as an anti-seizure therapy. This subject required a 20% warfarin dose reduction after CBD initiation and had no bleeding related adverse events. Damkier et al. (2019) report a 27-year-old male chronic polydrug substance user treated with warfarin for a mechanical heart valve replacement. After recreational cannabis smoking, his INR increased to 4.4 and returned to normal range after cessation. Hsu and colleagues (Hsu and Painter, 2020) report a 35-year-old male with a history of thrombosis, stable on warfarin for 8 years with a typical INR of 2.0–3.0. The subject started ingesting more cannabis products than usual for 1 month and his INR increased to 7.2. After withholding two doses of warfarin and cannabis cessation, his INR lowered to 3.0–4.0 without complications. Grayson and colleagues (Grayson et al., 2018) discuss a 44-year-old male with Marfan Syndrome, epilepsy, and mitral valve replacement requiring warfarin therapy. He had a stable INR of 2.0–2.6 for 6 months. His care team started escalating doses of CBD oil starting at 5 mg/kg/day and ending at 35 mg/kg/day after 17 months. During this period, his maintenance warfarin between visits started at 7.5 mg (0 mg/kg/day of CBD) and ended at 5.36 mg (35 mg/kg/day of CBD), an approximately 30% reduction. His INR range during this time was between 1.96 and 6.86. His care team monitored his INR during the cannabidiol administration, adjusted his warfarin, and no adverse events occurred. Brown et al. (2021) reported a 67-year-old man who took warfarin for deep vein thrombosis prevention, sublingual medical cannabis for chronic pain, and several other medications and supplements. He regularly took a total of 7.35 mg THC and 10.15 mg CBD per day. While counseled at the medical marijuana dispensary, he reported misunderstanding the dose instructions and scaled up his intake quickly to 14.7 mg THC and 10.3 mg CBD per day. After 3 days of the new regimen, his self-tested INR was 5.2 and his physician instructed him to skip his upcoming warfarin dose. He required a 29% dose reduction in warfarin to return INR to baseline and experienced mild dry mouth and transient dizziness, which subsided. Most recently, Thomas et al. (2022) reported an 85-year-old man taking warfarin for stroke prevention who started taking oromucosal oil formulations of cannabis for chronic low back pain. He was stable on 20–22 mg/week of warfarin and his cannabis regimen was 0.3 mg THC/5.3 mg CBD daily with 0.625 mg THC/0.625 mg CBD as needed. During the year of testing, his INR did not reach supratherapeutic ranges, and he was on a consistent dose of warfarin without adverse events. Serum testing of THC and CBD revealed estimated maximum concentrations of 0.35 and 0.87 ng/mL, respectively. The authors concluded that the THC and CBD concentrations were too low to significantly change the effects of warfarin for this patient.

### Tricyclic antidepressants (TCAs)

Two reports comprising five patients identified interactions between TCAs and cannabinoids which induced adverse events requiring emergent care. A case series by Wilens (1997) describes four male adolescents (ages 15–18) who smoked marijuana and were taking TCAs (nortriptyline, desipramine) for attention deficit hyperactivity disorder treatment. One patient was also taking clonidine, although no dose was reported. They experienced side effects ranging from confusion, lightheadedness, racing heart, and hallucinations–all potential effects of TCA toxicity. These adverse events were generally self-managed and abated after emergency department or home observation.

Another case report by Kizer (1980) details a 26-year-old male taking imipramine to treat “proctatosis”. This subject took an evening dose of imipramine and several hours later smoked a marijuana cigarette. He experienced disorientation, restlessness, dizziness, and heart palpitations, suggestive of TCA toxicity. After treatment with intramuscular injection of hydroxyzine, the patient’s symptoms abated.

### Transplant medications

We reviewed five studies (retrospective, case series, case reports) comprising 35 patients that report variable stability of serum levels of transplant medications due to ingestion of cannabinoids. Ebrahimi-Fakhari and colleagues (Ebrahimi-Fakhari et al., 2020) studied 25 patients who were treated with CBD and a mammalian Target Of Rapamycin (mTOR) inhibitor (18 everolimus, seven sirolimus). Serum mTOR inhibitor levels were significantly higher in 76% of patients after cannabidiol treatment. Some patients experienced doubling or tripling of their mTOR inhibitor trough serum level following cannabidiol, which resulted in clinical toxicity in 40% of patients (10/25). The most common adverse event was diarrhea, and there were no severe clinical toxicities. Additionally, some patients on phenytoin were found with higher-than-expected phenytoin levels, although this was not further explained. Cuñetti and colleagues (Cuñetti et al., 2018) report the effects of 21 days of scaled CBD on chronic pain and serum tacrolimus levels in seven kidney transplant cases. The authors report one patient requiring CBD dose reduction, three patients requiring tacrolimus titration and adverse events after CBD ingestion, and varying other side effects by patients including nausea, dry mouth, dizziness, and “heat episodes”. Three case reports also documented increases in tacrolimus blood levels following cannabis/cannabinoid use, two following edible formulations (gummies, lozenges) and another involving a CBD clinical trial. Hauser and colleagues (Hauser et al., 2016) documented serious tacrolimus toxicity in a 67-year-old male bone marrow transplant patient; drug levels were titrated to 8–12 ng/mL but spiked to 46 ng/mL and he was transferred to intensive care. He suffered from potential tacrolimus toxicity: diarrhea, stiffness, tremors, and altered mental status. The patient admitted to taking edible marijuana gummies prior to the tacrolimus blood level spike. After continued cannabis cessation, tacrolimus levels returned to normal, and treatment continued as planned. Moadel and Chism (2019) report a 48-year-old woman using tacrolimus post-liver transplant. Her dose was initially titrated to achieve a trough serum level 7.7 ng/mL, but unexpectedly this spiked to 17.2 ng/mL. She began exhibiting signs of encephalopathy secondary to tacrolimus toxicity with unclear etiology to the care team. The patient revealed a bottle of medical marijuana lozenges (10 mg THC:1 mg CBD per lozenge) and was taking two to four lozenges per day up until her transplant for pain control. After encouraging cannabis cessation, her tacrolimus level remained at goal. Leino and colleagues (Leino et al., 2019) report a 32-year-old woman taking tacrolimus for interstitial nephritis who entered a CBD clinical trial for epilepsy and showed an approximately 3-fold increase in previously dose-normalized tacrolimus plasma concentrations while receiving 2000–2,900 mg/day of CBD.

### Anticonvulsants

We have identified nine reports that report interactions between cannabinoids, specifically CBD, and narrow therapeutic index anti-epileptic drugs (AEDs). AEDs on the 57 NTI list include valproate, everolimus, and carbamazepine, among others. Three pharmacokinetic studies were published from the results of clinical trials sponsored by Jazz Pharmaceuticals to investigate the effects of drug-drug interactions involving CBD and antiepileptic drugs. Devinsky and colleagues (Devinsky et al., 2018) conducted a safety trial of 34 pediatric patients with Dravet syndrome taking CBD (5, 10, or 20 mg/kg) or placebo along with AEDs including valproate. Twenty patients of the CBD group experienced adverse events, and two needed to drop out of the study due to pyrexia, maculopapular rash, and elevated transaminase levels above criterion; the authors do not report which medications these patients were taking. Ben-Menachem et al. (2020) conducted a Phase 2, double-blind trial of 34 patients. Coadministration of cannabidiol with valproate produced lower effective serum concentrations of the drug and its metabolite (4-ene-VPA) in these patients: valproate exhibited a 17% decrease in AUC_tau_, and four-ene-VPA a 30% decrease in AUC_tau_. 14/16 subjects (87.5%) taking valproate experienced AEs with two discontinuing the trial because of adverse effects. A Phase 1 trial by Morrison et al. (2019) studied the effect of multiple dose administration of CBD on plasma concentrations of valproate and other AEDs in healthy subjects. They observed no relevant effect on valproate levels or pharmacokinetics. Nine subjects experienced rashes, five of which were involved with valproate administration with CBD; 4/5 of these subjects withdrew due to adverse events.

Two other trials of CBD and AED interactions have been conducted. An open label study by Gaston and colleagues (Gaston et al., 2017) reports drug-drug interactions between increasing cannabidiol (Epidiolex^®^) doses and 19 antiepileptic medications in 81 patients. Co-administration of CBD and valproate resulted in elevated liver function tests. The authors did not report the incidence of adverse events. Cáceres Guido et al. conducted a Phase 1 trial of 12 pediatric and adolescent patients concurrently taking up to 23 different AEDs, including valproate and levothyroxine, and induction of twice daily CBD oral administration or through a nasogastric (NG) tube (Cáceres Guido et al., 2021). CBD dosing started at 2 mg/kg/day and increased every 3 weeks. The two patients on levothyroxine experienced a 4-fold increase in CBD AUC_0-6_ compared to the rest of the cohort. No other pharmacokinetic changes were noted, and the authors did not study the serum medication levels of AEDs.

One retrospective chart review by McNamara and colleagues (McNamara et al., 2020) of pediatric patients suffering from epilepsy disorders and taking CBD compares laboratory abnormalities in those who took CBD with valproate (n = 26) and those who took CBD with another AED (n = 57). Those taking CBD and valproate concurrently had significantly higher incidence of thrombocytopenia (n = 9/26, 35%), defined as less than 110,000 platelets/μL in blood, compared to those who took CBD and another AED (n = 0/57, 0%, *p* < .0001). 4/9 (44%) of those with thrombocytopenia suffered ADRs such as easy bruising, hematuria (bloody urine), or gum bleeding. 8/9 (88%) required a dose adjustment or cessation of CBD or valproate, and all recovered. Additionally, those taking valproate and CBD had significantly higher levels of circulating liver enzymes (Aspartate Transaminase (AST) and Alanine Transaminase (ALT)), at 1-month (AST: *p* = .0009; ALT: *p* = .0001) and 3-month (AST: *p* = .003; ALT: *p* = .05) after starting concurrent therapy.

Klotz et al. (2019) reported a case series of five patients who were prescribed brivaracetam and other AEDs, including valproate and phenobarbital, and who administered increasing doses of CBD from 5 mg/kg/day to 25–50 mg/kg/day. The authors did not report any interactions nor adverse events with valproate or phenobarbital and CBD ingestion. Wiemer-Kruel and colleagues (Wiemer-Kruel et al., 2019) describe a 6-year-old female patient with Tuberous Sclerosis Complex related seizures prescribed everolimus and newly added adjunctive cannabidiol. After CBD initiation, serum everolimus levels were inconsistent (1.7–12.3 ug/L) despite consistent administered doses. Additionally, although the everolimus dose was halved, the trough levels quadrupled in the presence of CBD.

Ridout and authors describe a 37-year-old man with bipolar disorder (Ridout et al., 2021) experiencing varying levels of carbamazepine due to cannabis ingestion. He was titrated up to therapeutic levels of carbamazepine (1 g/day; serum level = 7.0 μg/mL), while smoking 1–2 marijuana cigarettes per day. After the patient discontinued cannabis, serum levels of carbamazepine dropped to 4.8 μg/mL, requiring a 20% increase in dose (1 g/day → 1.2 g/day). When the patient restarted 1 marijuana cigarette per day of cannabis use, carbamazepine levels were measured as supratherapeutic at 9.1 μg/mL and required reverting to the previous carbamazepine dose (1 g/day), after which serum levels returned to the therapeutic range (6.8 μg/mL).

### Quality of evidence assessment results

After excluding 18 case studies and series, 13 reports were assessed for quality of pharmacokinetic evidence. Three reports received a Good rating, nine received Fair, and one received Poor. Most studies had low sample sizes or did not thoroughly report cannabinoid use through dosing and frequency, both of which contributed to a lower quality score. 61.5% (*n* = 8/13) and 46.2% (*n* = 6/13) of the studies reported drug and cannabinoid pharmacokinetics, respectively. 61.5% (*n* = 8/13) included control subjects, and only 30.7% of studies (*n* = 4/13) included a balanced sex demographic distribution.

## Discussion

### *Cannabis* and CYP enzymes

As noted elsewhere, the cannabinoids are metabolized by the same cytochrome P-450 enzymes that are responsible for the majority of prescription drug metabolism (CYPs 3A4, 2C19 and 2C9) (Doohan et al., 2021). For that reason, they are prime candidates for altering prescription drug pharmacokinetics (Kocis and Vrana, 2020); our investigation also explored interactions with minor CYP enzymes as well as Phase II UGT enzymes. Our systematic review illuminated 18 out of 31 reports (58%) that either identified unexpected serum levels of prescription medications and/or the providers had to institute dose adjustments to optimize treatment or minimize side effects. In an inpatient setting with ample laboratory access, medications like tacrolimus, everolimus, and sirolimus levels can be titrated to avoid and mitigate under- and over-dosing of medications with sensitive therapeutic indices. This was seen in controlled settings where clinicians were initializing adjunctive CBD treatment. However, these instances are far from the norm of the general cannabis and cannabinoid user who typically consumes outside of the hospital and without the guidance of a clinician. This emphasizes the need for the clinician and patient to have an open dialog on the use of cannabinoids.

### Pharmacogenetics and pharmacogenomics

Naturally occurring variations in metabolizing enzymes is a topic of great interest to research and commercial scientists. In fact, the drug effect, reaction, and adherence of many antidepressant and antipsychotic medications are intertwined with their metabolism at key CYP enzymes (CYP2D6, 2C19, 3A4), and testing kits for clinicians and patients that analyze pharmacogenetic vulnerability and propensities have gained popularity to assist in treatment decisions (van Schaik et al., 2020). In addition, vulnerability to harmful outcomes of prescription or recreational drugs has been associated with selected CYP polymorphisms (Vevelstad et al., 2016; Rahikainen et al., 2018). Babayeva and colleagues provide an in-depth analysis of pharmacogenetic considerations in cannabinoid pharmacology and highlight conditions where altered function of these metabolizing enzymes creates potential for clinical harm (Mao et al., 2013; Babayeva and Loewy, 2023). This systematic review highlights the importance of understanding the interactions between cannabinoids and varying CYP and UGT enzymes in combination with drugs sensitive to altered metabolism.

### Adverse events as signals for drug interactions

While some clinicians may identify varying serum levels of narrow therapeutic index medications at the bedside, most clinicians encounter unexpected adverse reactions to prescribed medications as a signal to look for drug-drug interactions. When patients are prescribed NTI anticoagulants like warfarin and antidepressants like TCAs on an outpatient basis, varying cannabis use can alter metrics of treatment efficacy such as INR and mood, respectively. Conversely, in the perioperative setting, clinicians may not be able to rely on evidence-based algorithms for anesthetic requirements for medications like propofol if patients undergoing surgery have recently ingested cannabis or cannabinoids.

### Sex as a biological variable in cannabinoid drug-drug interactions

Sex contributes to the potential for drug-drug interactions and provides information that may be considered by healthcare professionals to reduce adverse events from prescribed medications and to optimize treatment response. Cannabinoids are highly lipophilic molecules with a non-selective pharmacodynamic profile and are metabolized by cytochrome P450 (CYP) enzymes. Each of these properties contributes to sex-dependent differences in potential drug interactions. First, lipophilicity is a factor in an ingested drug’s volume of distribution (Soldin and Mattison, 2009). Women tend to have a relatively higher body fat content than men, which increases the total storage capacity for lipophilic cannabinoids. Then, there is emerging research in sex-dependent cannabinoid receptor 1 (CB1) availability as measured by positron emission tomography (PET) radiotracer activity (Normandin et al., 2015). Finally, sex-dependent CYP enzyme activity and expression can influence the speed at which cannabinoids are metabolized as well as the relative competition between a cannabinoid and another medication (Soldin and Mattison, 2009). In a perfect research world, we would extract sex demographic data and incorporate the presence or absence of evenly distributed sex demographics. However, the majority of subjects studied in the included reports were male (59.8%), in agreement with the lack of evenly distributed samples in most clinical studies (Geller et al., 2018). While some diseases, that have approved or medicinally-purported cannabinoid remedies, also display sex biases (*i.e*., Lennox-Gestaut Syndrome has a male bias and multiple sclerosis has a female bias (Walton et al., 2020; Asadi-Pooya et al., 2021)), we did not identify any studies that reconciled sex bias or sex differences in recruitment strategies. None of the studies, stratified adverse events or pharmacological data by sex or hormonal status. This deficit in the literature offers opportunities for future clinical trials to incorporate sex and hormonal status into pharmacokinetic and safety analysis. Pharmacokinetic datasets including exogenous hormone replacement and hormonal status in any capacity are highly limited, and research on drug-drug interactions with variations in hormones is an emerging subject of study (Cirrincione et al., 2020; Sun et al., 2020).

### Meta-synthesis

Because of the heterogenous nature of the studies included in this review, a synthesis statement describing the findings of drug-drug interactions in each of the drug classes studied is provided; additionally, because the many drugs are used for widely different indications, our synthesis is stratified based on the intention of treatment within the study (*i.e*., anti-seizure were used to reduce epileptic activity and antidepressants were used to address depressive symptoms). This meta-synthesis is more appropriate than meta-analysis at the time of this review (given a lack of quantitative data). A meta-analysis of randomized controlled trials with standardized pharmacokinetic endpoints may be conducted in the future when enough trials exist.

For the methylxanthine derivatives, like theophylline, clearance of the drug is significantly increased compared to controls, requiring careful monitoring. Mismanaged theophylline levels can produce severe side effects including seizures, arrhythmias, and GI distress. In terms of analgesics and anesthetics, there is growing evidence for dynamic interactions between cannabis/cannabinoids and general anesthesia. A case report by Madden and colleagues provides a cautionary tale. This report describes a 13-year-old girl treated for pain with methadone (Madden et al., 2020), but the care team was unaware that her mother was supplementing her treatment with cannabidiol (250 mg orally, 6 times daily). She presented with extreme new onset fatigue and cannabidiol was discontinued. Her serum methadone levels were initially 271 ng/mL assessed 2 days after stopping CBD and this dropped to 149 mg/mL at day seven. By day 14, her fatigue returned to baseline and serum methadone was 125 mg/mL. Overall, four out of five studies examining propofol utilization report an increased dose required to induce anesthesia in cannabis users. Two other reports declared no difference in anesthetic requirements using propofol. One trial reported slight interactions with CBD clearance and fentanyl. Propofol requirements for patients who use cannabis should be further studied to improve dosing algorithms.

Warfarin, an anticoagulant, is currently one of the most widely prescribed medicines in the elderly, and unstable INRs (metric for blood thinning ability of warfarin) may produce adverse events. Six out of seven case reports (85.7%) describe variable INR when patients on warfarin ingested cannabis. 71.4% of cases (*n* = 5/7) required warfarin dose adjustment and 28.5% (*n* = 2/7) cases had adverse events. No clinical trials of anticoagulants and cannabis have been conducted. Patients on warfarin should be specifically queried on cannabis/cannabinoid use and counseled accordingly. Tricyclic antidepressants (TCAs), while typically prescribed for depressive psychiatric conditions, have a wide range of indications (on- and off-label) due to their non-selective activity at neurotransmitter systems across the brain and body. However, toxicity from higher-than-intended TCA levels includes a range of side effects, including dopaminergic and antimuscarinic instability. Two studies describe five patients who all suffered symptoms of TCA toxicity after ingesting cannabis. No serum levels of cannabinoids nor TCAs were drawn during these case studies, but caution must be taken in the concomitant use of TCAs and cannabis products.

Transplant medications (mTOR inhibitors like sirolimus and everolimus, and the calcineurin inhibitor tacrolimus) are used to treat some cancers, suppress immune responses to organ transplants, and prevent post-transplant complications due to immunoreactivity. We have identified one report describing interactions between cannabinoids and mTOR inhibitors and four reports describing interactions between cannabinoids and tacrolimus. Five articles, studying 35 patients undergoing treatment with tacrolimus, everolimus, and sirolimus, who concomitantly ingested THC and CBD formulations (sometimes under guidance of care team), reported varying levels of the prescription medication. In these studies, 46% (*n* = 16/35) of the patients suffered adverse events related to medication toxicity. Serum medication levels and co-ingestion of cannabinoids should therefore be carefully monitored. In a similar vein, anticonvulsant medications (also known as anti-epileptic drugs (AEDs)) are notoriously sensitive to drug-drug interactions due to heavy metabolism by the CYP450 system. Additionally, cannabinoid medications such as Epidiolex^®^ are seeing increasing utilization for seizure disorder therapy. Taken together, potentially severe DDIs exist between CBD and medications that are used in seizure disorders (everolimus [for tuberous sclerosis complex-associated seizures], valproic acid, and carbamazepine) or medications that can lower the seizure threshold (levothyroxine). Great care must be taken while using polypharmacy for seizure disorders as the nine identified studies in this area, comprising 332 patients, report 65 adverse events (19.6%).

### Limitations and future directions

This review was designed to extend our prediction that the metabolic characteristics of cannabinoids can be used to predict potential drug-drug interactions when there are common metabolizing enzymes for cannabinoids and prescription medications. Our results suggest that in recent history, a wide variety of adverse events and treatment inconsistencies arise when cannabinoids are co-administered with specific medications. A limitation for our work is the use of a list of theoretical interactions by narrow therapeutic index medications, which do not include other prescribed medications that can induce adverse events when taken with cannabinoids. Future systematic reviews may explore all drug-drug interactions and adverse events with cannabinoids, regardless of narrow therapeutic index status.

A significant limitation in all studies of these types is the lack of quality control in the composition of cannabis and cannabinoid products. Apart from the prescription cannabinoids (dronabinol, nabilone, Epidiolex^®^, or Sativex^®^ [nabiximols]), when patients admit to using a product, there is little or no information on the precise dose. That is, recreational cannabis comes with no information on the composition. The use of over-the-counter CBD oil also provides no information as demonstrated by a report by Hazekamp describing how commercial CBD products frequently contained much lower levels of the cannabinoid than indicated by the label (Hazekamp, 2018). Finally, the quality control of state-endorsed medical cannabinoid products varies widely.

## Conclusion

Given the metabolism of the cannabinoids (i.e., CBD and Δ^9^-THC) by the common cytochrome P-450 enzyme isoforms CYP3A4, CYP2C19, and CYP2C9, there should be no surprise that there is a great potential for drug-drug interactions. Indeed, we have identified 57 important medications that would theoretically interact with cannabis and cannabinoids (Kocis and Vrana, 2020). These prescription medications have the very real possibility of adverse drug reactions based on their narrow therapeutic index. In the present systematic review, we searched for clinical reports of real-world adverse drug events and/or dramatic changes in pharmacokinetics. This search identified 31 papers, evaluating more than 600 cannabinoid users, in which there was a direct link between cannabis or cannabinoid use and changes in prescription drug metabolism or therapeutic/toxic outcomes. As noted in Table 1, cannabinoid drug-drug interactions are not limited to any single class of medication. Instead, the concerns are driven by common metabolic enzymes. The interactions will reveal adverse events for those medications with narrow therapeutic indices.

Perhaps the most alarming aspects of cannabinoid drug-drug interactions and the potential for increased incidence are (a) the explosion of unregulated CBD oils in the marketplace; (b) the expansion of medical marijuana programs by individual states (many of which bypass the primary care physician); and (c) the decriminalization and reduced stigma of cannabis recreational use. These will, we believe, aggregate to increase the opportunity for cannabinoid drug-drug interactions. The large number of regional (U.S. states) and national programs for medical marijuana have created an ecosystem in which potent cannabinoid products can be taken for medical purposes, frequently without the involvement of a healthcare provider. As a result, pharmacodynamically active compounds can be added to the equation of prescription medications without consideration of the consequences. Finally, the growing landscape of legalized recreational cannabis further complicates matters as increased use of high THC-content products will be ingested without consideration of drug-drug interactions. We also note that these analyses have not considered the growing use of synthetic cannabinoids and delta-8-THC as recreational street drugs and cannabis adulterants.

In light of our concerns with potential drug-drug interactions and attendant adverse events, we have developed a freely available online tool for healthcare providers and patients (Kocis et al., 2023). CANN-DIR^®^ (CANNabinoid Drug Interaction Review; www.CANN-DIR.psu.edu) permits providers, caregivers, and patients the opportunity to enter their prescription medications and check for potential drug-drug interactions. This informational tool is available in ten different languages; however, at this time, the program does not highlight if there will be adverse events, but merely illuminates potential interactions for consideration.

In conclusion, this systematic review demonstrates real-world examples of cannabinoid drug-drug interactions with NTI prescription medications. While some of these interactions will not result in adverse events, it emphasizes the need for vigilance. Healthcare providers must probe, in a non-judgmental way, for use of recreational cannabis, medical cannabis products, and over-the-counter cannabinoid products (e.g., CBD and delta-8-THC).

## Data Availability

The original contributions presented in the study are included in the article/Supplementary Material, further inquiries can be directed to the corresponding author.
