# Supplementary material for: Systematic review of drug-drug interactions of delta-9-tetrahydrocannabinol, cannabidiol, and Cannabis
Source: Front Pharmacol. 2024 May 22;15:1282831. doi: 10.3389/fphar.2024.1282831 (PMC11167383; doi:10.3389/fphar.2024.1282831)
Supplement: Supplementary file 2 [file DataSheet1.docx]

**S1 Appendix – Search strategy for clinical drug-drug interactions with *Cannabis* or cannabinoids.**

**Antidepressant**

(((Dronabinol OR Medical Marijuana OR Marijuana Use OR Cannabidiol OR Cannabinoids OR Cannabaceae[MeSH Terms]) OR (Dronabinol [Title/Abstract] OR marinol[Title/Abstract] OR Syndros[Title/Abstract] OR Epidiolex[Title/Abstract] OR Nabilone [Title/Abstract] OR Cesamet[Title/Abstract] OR marijuana[Title/Abstract] OR Sativex[Title/Abstract] OR nabiximol[Title/Abstract] OR marihuana[Title/Abstract] OR Cannabidiol[Title/Abstract] OR cannabi*[Title/Abstract] OR cannabis[Title/Abstract] OR Cannabaceae[Title/Abstract] OR cannabigerolic[Title/Abstract] OR cannabichromene[Title/Abstract] OR Cannabigerol[Title/Abstract] OR delta-9-THC[Title/Abstract] OR Tetrahyrdocannabinol*[Title/Abstract] OR Cannabinoid*[Title/Abstract] OR delta(9)-THC[Title/Abstract] OR 9-ene-Tetrahydrocannabinol[Title/Abstract] OR Δ-9-tetrahydrocannabinol[Title/Abstract] OR delta(9)-Tetrahydrocannabinol[Title/Abstract] OR Tetrahydrocannabinol[Title/Abstract] OR 9-tetrahydrocannabinol[Title/Abstract] OR delta-9-tetrahydrocannabinol[Title/Abstract] OR tetra-hydrocannabinol*[Title/Abstract] OR sativ* OR indica[Title/Abstract] OR tetra-hydrocannabinol*[Title/Abstract] OR Tetrahydrocannabinol*[Title/Abstract]))) AND (((((((((((((((((((((((((((((((((((((((((((((((((((((((((((((((((((((((((((((((((((((((((((((((((((((((((((((((((((((((((((((((((((((((((((((amitriptyline[MeSH Terms]) OR amitriptyline[Title/Abstract]) OR amitid[Title/Abstract]) OR elavil[Title/Abstract]) OR amitril[Title/Abstract]) OR endep[Title/Abstract]) OR vanatrip[Title/Abstract]) OR amitrip[Title/Abstract]) OR clomipramine[MeSH Terms]) OR clomipramine[Title/Abstract]) OR anafranil[Title/Abstract]) OR Apo-clomiPRAMINE[Title/Abstract]) OR clofranil[Title/Abstract]) OR Clomicalm[Title/Abstract]) OR Clomidep[Title/Abstract]) OR clomiPRAMINE[Title/Abstract]) OR clomipran[Title/Abstract]) OR clopram[Title/Abstract]) OR Co clomipramine[Title/Abstract]) OR denapranil[Title/Abstract]) OR equinorm[Title/Abstract]) OR fenatil[Title/Abstract]) OR hydiphen[Title/Abstract]) OR maronil[Title/Abstract]) OR Novo-Clopamine[Title/Abstract]) OR placil[Title/Abstract]) OR qualifranil[Title/Abstract]) OR tranquax[Title/Abstract]) OR zoiral[Title/Abstract]) OR desipramine[MeSH Terms]) OR desipramine[Title/Abstract]) OR norpramin[Title/Abstract]) OR Apo-Desipramine[Title/Abstract]) OR deprexan[Title/Abstract]) OR desipramine[Title/Abstract]) OR distonal[Title/Abstract]) OR norpramin[Title/Abstract]) OR nortimil[Title/Abstract]) OR pertofran[Title/Abstract]) OR petylyl[Title/Abstract]) OR sertofren[Title/Abstract]) OR dosulepin[Title/Abstract]) OR dosulepina[Title/Abstract]) OR dosulepine[Title/Abstract]) OR dosulepinum[Title/Abstract]) OR dothiepin[Title/Abstract]) OR depropin[Title/Abstract]) OR Dopin[Title/Abstract]) OR Dopress[Title/Abstract]) OR Dothapax[Title/Abstract]) OR Dothcin[Title/Abstract]) OR Dothep[Title/Abstract]) OR Dothip[Title/Abstract]) OR Espin[Title/Abstract]) OR Harmomed[Title/Abstract]) OR Idom[Title/Abstract]) OR Jardin[Title/Abstract]) OR Othtric[Title/Abstract]) OR Prepadine[Title/Abstract]) OR Prothiaden[Title/Abstract]) OR Qualiaden[Title/Abstract]) OR Thaden[Title/Abstract]) OR Vick-Thiaden[Title/Abstract]) OR Harmomed, Idom, Jardin,Othtric,Prepadine,Prothiaden, Protiaden , Protiadene, Qualiaden, Thaden,Thaden, Vick-Thiaden, Xerenal[Title/Abstract]) OR Xerenal[Title/Abstract]) OR doxepin[MeSH Terms]) OR doxepin[Title/Abstract]) OR Prudoxin[Title/Abstract]) OR SINEquan[Title/Abstract]) OR adapin[Title/Abstract]) OR antimax[Title/Abstract]) OR Apo-Doxepin[Title/Abstract]) OR Aponal[Title/Abstract]) OR Deptran[Title/Abstract]) OR Desidoxepin[Title/Abstract]) OR Doneurin[Title/Abstract]) OR Doxal[Title/Abstract]) OR doxe[Title/Abstract]) OR doxepia[Title/Abstract]) OR doxepin[Title/Abstract]) OR Sinequan[Title/Abstract]) OR Triadapin[Title/Abstract]) OR Xepin[Title/Abstract]) OR Zonalon[Title/Abstract]) OR Prudoxin[Title/Abstract]) OR Quitaxon[Title/Abstract]) OR Silenor[Title/Abstract]) OR esketamine[Title/Abstract]) OR Spravato[Title/Abstract]) OR Esketiv[Title/Abstract]) OR Ketanest[Title/Abstract]) OR imipramine[MeSH Terms]) OR imipramine[Title/Abstract]) OR tofranil[Title/Abstract]) OR Antidep[Title/Abstract]) OR Antipress[Title/Abstract]) OR Antideprin[Title/Abstract]) OR Apo-Imipramine[Title/Abstract]) OR Celamine[Title/Abstract]) OR Chrytemin[Title/Abstract]) OR Depramina[Title/Abstract]) OR Ethipramine[Title/Abstract]) OR Imavate[Title/Abstract]) OR Imine[Title/Abstract]) OR Imipra[Title/Abstract]) OR Imipramine[Title/Abstract]) OR Imiprex[Title/Abstract]) OR Imiprin[Title/Abstract]) OR Impril[Title/Abstract]) OR Janimine[Title/Abstract]) OR Medipramine[Title/Abstract]) OR Norfranil[Title/Abstract]) OR Novo-Pramine[Title/Abstract]) OR Paidenur[Title/Abstract]) OR Praminan[Title/Abstract]) OR Presamine[Title/Abstract]) OR Primonil[Title/Abstract]) OR Pryleugan[Title/Abstract]) OR Sermonil[Title/Abstract]) OR Sk-Pramine[Title/Abstract]) OR Surplix[Title/Abstract]) OR Talpramin[Title/Abstract]) OR Tofranil[Title/Abstract]) OR lofepramine[MeSH Terms]) OR lofepramine[Title/Abstract]) OR Gamanil[Title/Abstract]) OR Lomont[Title/Abstract]) OR Tymelyt[Title/Abstract]) OR lofépramine[Title/Abstract]) OR Amplit[Title/Abstract]) OR Deftan[Title/Abstract]) OR Deprimil[Title/Abstract]) OR Emdalen[Title/Abstract]) OR Gamonil[Title/Abstract]) OR Tymelet[Title/Abstract]) OR "hydrochloride salt"[Title/Abstract]) OR Amplit[Title/Abstract]) OR nortriptyline[Title/Abstract]) OR Lopramine[Title/Abstract]) OR tianeptine[Title/Abstract]) OR trimipramine[Title/Abstract])

**Antibiotic**

(((((Dronabinol OR Medical Marijuana OR Marijuana Use OR Cannabidiol OR Cannabinoids OR Cannabaceae[MeSH Terms]) OR (Dronabinol [Title/Abstract] OR marinol[Title/Abstract] OR Syndros[Title/Abstract] OR Epidiolex[Title/Abstract] OR Nabilone [Title/Abstract] OR Cesamet[Title/Abstract] OR marijuana[Title/Abstract] OR Sativex[Title/Abstract] OR nabiximol[Title/Abstract] OR marihuana[Title/Abstract] OR Cannabidiol[Title/Abstract] OR cannabi*[Title/Abstract] OR cannabis[Title/Abstract] OR Cannabaceae[Title/Abstract] OR cannabigerolic[Title/Abstract] OR cannabichromene[Title/Abstract] OR Cannabigerol[Title/Abstract] OR delta-9-THC[Title/Abstract] OR Tetrahyrdocannabinol*[Title/Abstract] OR Cannabinoid*[Title/Abstract] OR delta(9)-THC[Title/Abstract] OR 9-ene-Tetrahydrocannabinol[Title/Abstract] OR Δ-9-tetrahydrocannabinol[Title/Abstract] OR delta(9)-Tetrahydrocannabinol[Title/Abstract] OR Tetrahydrocannabinol[Title/Abstract] OR 9-tetrahydrocannabinol[Title/Abstract] OR delta-9-tetrahydrocannabinol[Title/Abstract] OR tetra-hydrocannabinol*[Title/Abstract] OR sativ* OR indica[Title/Abstract] OR tetra-hydrocannabinol*[Title/Abstract] OR Tetrahydrocannabinol*[Title/Abstract]))) OR (Bediol OR Bedrocan))) AND ((("Clindamycin"[Mesh]) OR "Amphotericin B"[Mesh]) OR ((((((((((amphotericin[Title/Abstract]) OR Amphocin[Title/Abstract]) OR Fungizone[Title/Abstract]) OR Cleocin[Title/Abstract]) OR clindamycin[Title/Abstract]) OR Chlolincocin[Title/Abstract]) OR Clindamyc*[Title/Abstract]) OR Klimicin[Title/Abstract]) OR Dalacin) OR Copal))

**Antipsychotic**

((((((Pimozide[Title/Abstract]) OR Orap[Title/Abstract]) OR Antalon[Title/Abstract])) OR "Pimozide"[Mesh])) AND ((((Dronabinol OR Medical Marijuana OR Marijuana Use OR Cannabidiol OR Cannabinoids OR Cannabaceae[MeSH Terms]) OR (Dronabinol [Title/Abstract] OR marinol[Title/Abstract] OR Syndros[Title/Abstract] OR Epidiolex[Title/Abstract] OR Nabilone [Title/Abstract] OR Cesamet[Title/Abstract] OR marijuana[Title/Abstract] OR Sativex[Title/Abstract] OR nabiximol[Title/Abstract] OR marihuana[Title/Abstract] OR Cannabidiol[Title/Abstract] OR cannabi*[Title/Abstract] OR cannabis[Title/Abstract] OR Cannabaceae[Title/Abstract] OR cannabigerolic[Title/Abstract] OR cannabichromene[Title/Abstract] OR Cannabigerol[Title/Abstract] OR delta-9-THC[Title/Abstract] OR Tetrahyrdocannabinol*[Title/Abstract] OR Cannabinoid*[Title/Abstract] OR delta(9)-THC[Title/Abstract] OR 9-ene-Tetrahydrocannabinol[Title/Abstract] OR Δ-9-tetrahydrocannabinol[Title/Abstract] OR delta(9)-Tetrahydrocannabinol[Title/Abstract] OR Tetrahydrocannabinol[Title/Abstract] OR 9-tetrahydrocannabinol[Title/Abstract] OR delta-9-tetrahydrocannabinol[Title/Abstract] OR tetra-hydrocannabinol*[Title/Abstract] OR sativ* OR indica[Title/Abstract] OR tetra-hydrocannabinol*[Title/Abstract] OR Tetrahydrocannabinol*[Title/Abstract])))

**Anticoagulant**

(((Dronabinol OR Medical Marijuana OR Marijuana Use OR Cannabidiol OR Cannabinoids OR Cannabaceae[MeSH Terms]) OR (Dronabinol [Title/Abstract] OR marinol[Title/Abstract] OR Syndros[Title/Abstract] OR Epidiolex[Title/Abstract] OR Nabilone [Title/Abstract] OR Cesamet[Title/Abstract] OR marijuana[Title/Abstract] OR Sativex[Title/Abstract] OR nabiximol[Title/Abstract] OR marihuana[Title/Abstract] OR Cannabidiol[Title/Abstract] OR cannabi*[Title/Abstract] OR cannabis[Title/Abstract] OR Cannabaceae[Title/Abstract] OR cannabigerolic[Title/Abstract] OR cannabichromene[Title/Abstract] OR Cannabigerol[Title/Abstract] OR delta-9-THC[Title/Abstract] OR Tetrahyrdocannabinol*[Title/Abstract] OR Cannabinoid*[Title/Abstract] OR delta(9)-THC[Title/Abstract] OR 9-ene-Tetrahydrocannabinol[Title/Abstract] OR Δ-9-tetrahydrocannabinol[Title/Abstract] OR delta(9)-Tetrahydrocannabinol[Title/Abstract] OR Tetrahydrocannabinol[Title/Abstract] OR 9-tetrahydrocannabinol[Title/Abstract] OR delta-9-tetrahydrocannabinol[Title/Abstract] OR tetra-hydrocannabinol*[Title/Abstract] OR sativ* OR indica[Title/Abstract] OR tetra-hydrocannabinol*[Title/Abstract] OR Tetrahydrocannabinol*[Title/Abstract]))) AND (((((((((dabigatran[Title/Abstract]) OR argatroban[Title/Abstract]) OR Acova[Title/Abstract]) OR dabigatran[Title/Abstract]) OR Pradaxa[Title/Abstract]) OR BIBR 1048[Title/Abstract])) OR "Dabigatran"[Mesh]) OR "argatroban" [Supplementary Concept])

**Anticoagulant**

(((Dronabinol OR Medical Marijuana OR Marijuana Use OR Cannabidiol OR Cannabinoids OR Cannabaceae[MeSH Terms]) OR (Dronabinol [Title/Abstract] OR marinol[Title/Abstract] OR Syndros[Title/Abstract] OR Epidiolex[Title/Abstract] OR Nabilone [Title/Abstract] OR Cesamet[Title/Abstract] OR marijuana[Title/Abstract] OR Sativex[Title/Abstract] OR nabiximol[Title/Abstract] OR marihuana[Title/Abstract] OR Cannabidiol[Title/Abstract] OR cannabi*[Title/Abstract] OR cannabis[Title/Abstract] OR Cannabaceae[Title/Abstract] OR cannabigerolic[Title/Abstract] OR cannabichromene[Title/Abstract] OR Cannabigerol[Title/Abstract] OR delta-9-THC[Title/Abstract] OR Tetrahyrdocannabinol*[Title/Abstract] OR Cannabinoid*[Title/Abstract] OR delta(9)-THC[Title/Abstract] OR 9-ene-Tetrahydrocannabinol[Title/Abstract] OR Δ-9-tetrahydrocannabinol[Title/Abstract] OR delta(9)-Tetrahydrocannabinol[Title/Abstract] OR Tetrahydrocannabinol[Title/Abstract] OR 9-tetrahydrocannabinol[Title/Abstract] OR delta-9-tetrahydrocannabinol[Title/Abstract] OR tetra-hydrocannabinol*[Title/Abstract] OR sativ* OR indica[Title/Abstract] OR tetra-hydrocannabinol*[Title/Abstract] OR Tetrahydrocannabinol*[Title/Abstract]))) AND (((((((((dabigatran[Title/Abstract]) OR argatroban[Title/Abstract]) OR Acova[Title/Abstract]) OR dabigatran[Title/Abstract]) OR Pradaxa[Title/Abstract]) OR BIBR 1048[Title/Abstract])) OR "Dabigatran"[Mesh]) OR "argatroban" [Supplementary Concept])

**Asthma**

(((((aminophylline[MeSH Terms]) OR Theophylline[MeSH Terms])) OR (((((((((((((((Theophylline[Title/Abstract]) OR Aminophylline[Title/Abstract]) OR Norphyl[Title/Abstract]) OR Phyllocontin[Title/Abstract]) OR Truphylline[Title/Abstract]) OR Theophylline[Title/Abstract]) OR Afonilum[Title/Abstract]) OR Aerolate[Title/Abstract]) OR Uniphyl[Title/Abstract]) OR Theo-Dur[Title/Abstract]) OR Theo-Time[Title/Abstract]) OR TheoCap[Title/Abstract]) OR Theochron[Title/Abstract]) OR Elixophyllin[Title/Abstract]) OR Quibron-T[Title/Abstract]))) AND ((((Dronabinol OR Medical Marijuana OR Marijuana Use OR Cannabidiol OR Cannabinoids OR Cannabaceae[MeSH Terms]) OR (Dronabinol [Title/Abstract] OR marinol[Title/Abstract] OR Syndros[Title/Abstract] OR Epidiolex[Title/Abstract] OR Nabilone [Title/Abstract] OR Cesamet[Title/Abstract] OR marijuana[Title/Abstract] OR Sativex[Title/Abstract] OR nabiximol[Title/Abstract] OR marihuana[Title/Abstract] OR Cannabidiol[Title/Abstract] OR cannabi*[Title/Abstract] OR cannabis[Title/Abstract] OR Cannabaceae[Title/Abstract] OR cannabigerolic[Title/Abstract] OR cannabichromene[Title/Abstract] OR Cannabigerol[Title/Abstract] OR delta-9-THC[Title/Abstract] OR Tetrahyrdocannabinol*[Title/Abstract] OR Cannabinoid*[Title/Abstract] OR delta(9)-THC[Title/Abstract] OR 9-ene-Tetrahydrocannabinol[Title/Abstract] OR Δ-9-tetrahydrocannabinol[Title/Abstract] OR delta(9)-Tetrahydrocannabinol[Title/Abstract] OR Tetrahydrocannabinol[Title/Abstract] OR 9-tetrahydrocannabinol[Title/Abstract] OR delta-9-tetrahydrocannabinol[Title/Abstract] OR tetra-hydrocannabinol*[Title/Abstract] OR sativ* OR indica[Title/Abstract] OR tetra-hydrocannabinol*[Title/Abstract] OR Tetrahydrocannabinol*[Title/Abstract])))

**Antiarrhythmic**

(((((Dronabinol OR Medical Marijuana OR Marijuana Use OR Cannabidiol OR Cannabinoids OR Cannabaceae[MeSH Terms]) OR (Dronabinol [Title/Abstract] OR marinol[Title/Abstract] OR Syndros[Title/Abstract] OR Epidiolex[Title/Abstract] OR Nabilone [Title/Abstract] OR Cesamet[Title/Abstract] OR marijuana[Title/Abstract] OR Sativex[Title/Abstract] OR nabiximol[Title/Abstract] OR marihuana[Title/Abstract] OR Cannabidiol[Title/Abstract] OR cannabi*[Title/Abstract] OR cannabis[Title/Abstract] OR Cannabaceae[Title/Abstract] OR cannabigerolic[Title/Abstract] OR cannabichromene[Title/Abstract] OR Cannabigerol[Title/Abstract] OR delta-9-THC[Title/Abstract] OR Tetrahyrdocannabinol*[Title/Abstract] OR Cannabinoid*[Title/Abstract] OR delta(9)-THC[Title/Abstract] OR 9-ene-Tetrahydrocannabinol[Title/Abstract] OR Δ-9-tetrahydrocannabinol[Title/Abstract] OR delta(9)-Tetrahydrocannabinol[Title/Abstract] OR Tetrahydrocannabinol[Title/Abstract] OR 9-tetrahydrocannabinol[Title/Abstract] OR delta-9-tetrahydrocannabinol[Title/Abstract] OR tetra-hydrocannabinol*[Title/Abstract] OR sativ* OR indica[Title/Abstract] OR tetra-hydrocannabinol*[Title/Abstract] OR Tetrahydrocannabinol*[Title/Abstract]))) OR (Bediol OR Bedrocan))) AND ((((("Amiodarone"[Mesh]) OR "Digitoxin"[Mesh]) OR "dofetilide" [Supplementary Concept])) OR ((((((((((((((((Amiodar*[Title/Abstract]) OR Cordarone[Title/Abstract]) OR Pacerone[Title/Abstract]) OR Nexterone[Title/Abstract]) OR Aratac[Title/Abstract]) OR Digitoxin[Title/Abstract]) OR Digitaline[Title/Abstract]) OR Digophton[Title/Abstract]) OR Dofetilide[Title/Abstract]) OR Tikosyn[Title/Abstract]) OR Quinidine[Title/Abstract]) OR Quinaglute[Title/Abstract]) OR Quinalan[Title/Abstract]) OR Quinidex[Title/Abstract]) OR Chinidin[Title/Abstract]) OR Quincardine[Title/Abstract]))

**Analgesic**

(((((((((((((((((((((((((((((((((((((((((((((((((((((((((alfentanil[MeSH Terms]) OR fentanyl[MeSH Terms]) OR meperidine[MeSH Terms]) OR operidine[MeSH Terms]) OR Propofol[MeSH Terms]) OR Thiopental[MeSH Terms]) OR alfentanil[Title/Abstract]) OR Alfenta[Title/Abstract]) OR Limifen[Title/Abstract]) OR Rapifen[Title/Abstract]) OR Fanaxal[Title/Abstract]) OR Alfentanilum[Title/Abstract]) OR Alfentanyl[Title/Abstract]) OR fentanyl[Title/Abstract]) OR Duragesic[Title/Abstract]) OR Subsys[Title/Abstract]) OR Ionsys[Title/Abstract]) OR Duragesic[Title/Abstract]) OR Fentanil[Title/Abstract]) OR Phentanyl[Title/Abstract]) OR Sublimaze[Title/Abstract]) OR meperidine[Title/Abstract]) OR Pethidine[Title/Abstract]) OR Isonipecain[Title/Abstract]) OR Dolsin[Title/Abstract]) OR Dolosal[Title/Abstract]) OR Dolin[Title/Abstract]) OR Operidine[Title/Abstract]) OR Dolantin[Title/Abstract]) OR Dolargan[Title/Abstract]) OR Demerol[Title/Abstract]) OR Meperitab[Title/Abstract]) OR Operidine[Title/Abstract]) OR Phenoperidine[Title/Abstract]) OR Lealgin[Title/Abstract]) OR Diamorphine[Title/Abstract]) OR Propofol[Title/Abstract]) OR Diprivan[Title/Abstract]) OR Aquafo[Title/Abstract]) OR Diisopropylphenol[Title/Abstract]) OR Diprifusor[Title/Abstract]) OR Diprivan[Title/Abstract]) OR Disoprivan[Title/Abstract]) OR Pofol[Title/Abstract]) OR Disoprofol[Title/Abstract]) OR Propofolum[Title/Abstract]) OR Thiopental[Title/Abstract]) OR Pentothal[Title/Abstract]) OR thiopental[Title/Abstract]) OR thiopentone[Title/Abstract]) OR Trapanal[Title/Abstract]) OR Thiomebumal[Title/Abstract]) OR Thiopentone[Title/Abstract]) OR Thiopentobarbital[Title/Abstract]) OR Thionembutal[Title/Abstract]) AND Humans[Mesh])) AND (((((Dronabinol OR Medical Marijuana OR Marijuana Use OR Cannabidiol OR Cannabinoids OR Cannabaceae[MeSH Terms]) OR (Dronabinol [Title/Abstract] OR marinol[Title/Abstract] OR Syndros[Title/Abstract] OR Epidiolex[Title/Abstract] OR Nabilone[Title/Abstract] OR Cesamet[Title/Abstract] OR marijuana[Title/Abstract] OR Sativex[Title/Abstract] OR nabiximols[Title/Abstract] OR marihuana[Title/Abstract] OR Cannabidiol[Title/Abstract] OR cannabi*[Title/Abstract] OR cannabis[Title/Abstract] cannabis[Title/Abstract] OR Cannabaceae[Title/Abstract] OR cannabigerolic[Title/Abstract] OR cannabichromene[Title/Abstract] OR Cannabigerol[Title/Abstract] OR delta-9-THC[Title/Abstract] OR Tetrahydrocannabinol*[Title/Abstract] OR Cannabinoid*[Title/Abstract] OR delta(9)-THC[Title/Abstract] OR 9-ene-Tetrahydrocannabinol[Title/Abstract] OR Δ-9-tetrahydrocannabinol[Title/Abstract] OR delta(9)-Tetrahydrocannabinol[Title/Abstract] OR Tetrahydrocannabinol[Title/Abstract] OR 9-tetrahydrocannabinol[Title/Abstract] OR delta-9-tetrahydrocannabinol[Title/Abstract] OR tetra-hydrocannabinol*[Title/Abstract] OR sativ* OR indica[Title/Abstract] OR tetra-hydrocannabinol*[Title/Abstract] OR Tetrahydrocannabinol*[Title/Abstract])))

**Experimental/unknown**

(((Dronabinol OR Medical Marijuana OR Marijuana Use OR Cannabidiol OR Cannabinoids OR Cannabaceae[MeSH Terms]) OR (Dronabinol [Title/Abstract] OR marinol[Title/Abstract] OR Syndros[Title/Abstract] OR Epidiolex[Title/Abstract] OR Nabilone [Title/Abstract] OR Cesamet[Title/Abstract] OR marijuana[Title/Abstract] OR Sativex[Title/Abstract] OR nabiximol[Title/Abstract] OR marihuana[Title/Abstract] OR Cannabidiol[Title/Abstract] OR cannabi*[Title/Abstract] OR cannabis[Title/Abstract] OR Cannabaceae[Title/Abstract] OR cannabigerolic[Title/Abstract] OR cannabichromene[Title/Abstract] OR Cannabigerol[Title/Abstract] OR delta-9-THC[Title/Abstract] OR Tetrahyrdocannabinol*[Title/Abstract] OR Cannabinoid*[Title/Abstract] OR delta(9)-THC[Title/Abstract] OR 9-ene-Tetrahydrocannabinol[Title/Abstract] OR Δ-9-tetrahydrocannabinol[Title/Abstract] OR delta(9)-Tetrahydrocannabinol[Title/Abstract] OR Tetrahydrocannabinol[Title/Abstract] OR 9-tetrahydrocannabinol[Title/Abstract] OR delta-9-tetrahydrocannabinol[Title/Abstract] OR tetra-hydrocannabinol*[Title/Abstract] OR sativ* OR indica[Title/Abstract] OR tetra-hydrocannabinol*[Title/Abstract] OR Tetrahydrocannabinol*[Title/Abstract]))) AND ((((Melitracen[MeSH Terms]) OR Melitracen[Title/Abstract]) OR flupentixol[Title/Abstract]) OR Deanxit[Title/Abstract])

**Thyroid**

(((((((((Levothyroxine[MeSH Terms]) OR Levothyroxine[Title/Abstract]) OR Levothroid[Title/Abstract]) OR Levoxyl[Title/Abstract]) OR Synthroid[Title/Abstract]) OR Unithroid[Title/Abstract]) OR Tirosint[Title/Abstract]) OR Tirosint-Sol[Title/Abstract])) AND ((Dronabinol OR Medical Marijuana OR Marijuana Use OR Cannabidiol OR Cannabinoids OR Cannabaceae[MeSH Terms]) OR (Dronabinol [Title/Abstract] OR marinol[Title/Abstract] OR Syndros[Title/Abstract] OR Epidiolex[Title/Abstract] OR Nabilone [Title/Abstract] OR Cesamet[Title/Abstract] OR marijuana[Title/Abstract] OR Sativex[Title/Abstract] OR nabiximol[Title/Abstract] OR marihuana[Title/Abstract] OR Cannabidiol[Title/Abstract] OR cannabi*[Title/Abstract] OR cannabis[Title/Abstract] OR Cannabaceae[Title/Abstract] OR cannabigerolic[Title/Abstract] OR cannabichromene[Title/Abstract] OR Cannabigerol[Title/Abstract] OR delta-9-THC[Title/Abstract] OR Tetrahyrdocannabinol*[Title/Abstract] OR Cannabinoid*[Title/Abstract] OR delta(9)-THC[Title/Abstract] OR 9-ene-Tetrahydrocannabinol[Title/Abstract] OR Δ-9-tetrahydrocannabinol[Title/Abstract] OR delta(9)-Tetrahydrocannabinol[Title/Abstract] OR Tetrahydrocannabinol[Title/Abstract] OR 9-tetrahydrocannabinol[Title/Abstract] OR delta-9-tetrahydrocannabinol[Title/Abstract] OR tetra-hydrocannabinol*[Title/Abstract] OR sativ* OR indica[Title/Abstract] OR tetra-hydrocannabinol*[Title/Abstract] OR Tetrahydrocannabinol*[Title/Abstract]))

**Oral Contraceptives**

((((((((((((Ethinyl Estradiol[MeSH Terms]) OR Estradiol[MeSH Terms]) OR Estradiol[Title/Abstract]) OR Ethinylestradiol[Title/Abstract]) OR Aethinyloestradiolum[Title/Abstract]) OR Ethiny[Title/Abstract]) OR Ethinyloestradiol[Title/Abstract]) OR Etynyloestradiol[Title/Abstract]) OR Progynon[Title/Abstract]) OR Estinyl[Title/Abstract]) OR Oestradio[Title/Abstract])) AND ((Dronabinol OR Medical Marijuana OR Marijuana Use OR Cannabidiol OR Cannabinoids OR Cannabaceae[MeSH Terms]) OR (Dronabinol [Title/Abstract] OR marinol[Title/Abstract] OR Syndros[Title/Abstract] OR Epidiolex[Title/Abstract] OR Nabilone [Title/Abstract] OR Cesamet[Title/Abstract] OR marijuana[Title/Abstract] OR Sativex[Title/Abstract] OR nabiximol[Title/Abstract] OR marihuana[Title/Abstract] OR Cannabidiol[Title/Abstract] OR cannabi*[Title/Abstract] OR cannabis[Title/Abstract] OR Cannabaceae[Title/Abstract] OR cannabigerolic[Title/Abstract] OR cannabichromene[Title/Abstract] OR Cannabigerol[Title/Abstract] OR delta-9-THC[Title/Abstract] OR Tetrahyrdocannabinol*[Title/Abstract] OR Cannabinoid*[Title/Abstract] OR delta(9)-THC[Title/Abstract] OR 9-ene-Tetrahydrocannabinol[Title/Abstract] OR Δ-9-tetrahydrocannabinol[Title/Abstract] OR delta(9)-Tetrahydrocannabinol[Title/Abstract] OR Tetrahydrocannabinol[Title/Abstract] OR 9-tetrahydrocannabinol[Title/Abstract] OR delta-9-tetrahydrocannabinol[Title/Abstract] OR tetra-hydrocannabinol*[Title/Abstract] OR sativ* OR indica[Title/Abstract] OR tetra-hydrocannabinol*[Title/Abstract] OR Tetrahydrocannabinol*[Title/Abstract]))

**Migraines**

((dihydroergotamine[Title/Abstract] OR Migranal[Title/Abstract] OR Embolex[Title/Abstract] OR Levadex[Title/Abstract] OR ergotamine[Title/Abstract] OR Ergomar[Title/Abstract] OR Ergotamin[Title/Abstract] OR Gynergen[Title/Abstract] OR Cafergot[Title/Abstract] OR "valproic acid"[Title/Abstract] OR Depakote[Title/Abstract] OR Epilim[Title/Abstract] OR Convulex[Title/Abstract] OR "Depakene Stavzor"[Title/Abstract] OR Depakin[Title/Abstract] OR Depakine[Title/Abstract] OR Ergenyl[Title/Abstract] OR Valproate[Title/Abstract] OR Vupral[Title/Abstract]) OR ((("Dihydroergotamine"[Mesh]) OR "Ergotamine"[Mesh]) OR "Valproic Acid"[Mesh])) AND ((((Dronabinol OR Medical Marijuana OR Marijuana Use OR Cannabidiol OR Cannabinoids OR Cannabaceae[MeSH Terms]) OR (Dronabinol [Title/Abstract] OR marinol[Title/Abstract] OR Syndros[Title/Abstract] OR Epidiolex[Title/Abstract] OR Nabilone [Title/Abstract] OR Cesamet[Title/Abstract] OR marijuana[Title/Abstract] OR Sativex[Title/Abstract] OR nabiximol[Title/Abstract] OR marihuana[Title/Abstract] OR Cannabidiol[Title/Abstract] OR cannabi*[Title/Abstract] OR cannabis[Title/Abstract] OR Cannabaceae[Title/Abstract] OR cannabigerolic[Title/Abstract] OR cannabichromene[Title/Abstract] OR Cannabigerol[Title/Abstract] OR delta-9-THC[Title/Abstract] OR Tetrahyrdocannabinol*[Title/Abstract] OR Cannabinoid*[Title/Abstract] OR delta(9)-THC[Title/Abstract] OR 9-ene-Tetrahydrocannabinol[Title/Abstract] OR Δ-9-tetrahydrocannabinol[Title/Abstract] OR delta(9)-Tetrahydrocannabinol[Title/Abstract] OR Tetrahydrocannabinol[Title/Abstract] OR 9-tetrahydrocannabinol[Title/Abstract] OR delta-9-tetrahydrocannabinol[Title/Abstract] OR tetra-hydrocannabinol*[Title/Abstract] OR sativ* OR indica[Title/Abstract] OR tetra-hydrocannabinol*[Title/Abstract] OR Tetrahydrocannabinol*[Title/Abstract])))

**Elevated Blood Pressure**

(((((((((Clonidine[MeSH Terms]) OR Clonidin*[Title/Abstract]) OR Catapres[Title/Abstract]) OR Kapvay[Title/Abstract]) OR Nexiclon[Title/Abstract]) OR Duraclon[Title/Abstract]) OR Kapvay[Title/Abstract]) OR Catapres[Title/Abstract])) AND ((((Dronabinol OR Medical Marijuana OR Marijuana Use OR Cannabidiol OR Cannabinoids OR Cannabaceae[MeSH Terms]) OR (Dronabinol [Title/Abstract] OR marinol[Title/Abstract] OR Syndros[Title/Abstract] OR Epidiolex[Title/Abstract] OR Nabilone [Title/Abstract] OR Cesamet[Title/Abstract] OR marijuana[Title/Abstract] OR Sativex[Title/Abstract] OR nabiximol[Title/Abstract] OR marihuana[Title/Abstract] OR Cannabidiol[Title/Abstract] OR cannabi*[Title/Abstract] OR cannabis[Title/Abstract] OR Cannabaceae[Title/Abstract] OR cannabigerolic[Title/Abstract] OR cannabichromene[Title/Abstract] OR Cannabigerol[Title/Abstract] OR delta-9-THC[Title/Abstract] OR Tetrahyrdocannabinol*[Title/Abstract] OR Cannabinoid*[Title/Abstract] OR delta(9)-THC[Title/Abstract] OR 9-ene-Tetrahydrocannabinol[Title/Abstract] OR Δ-9-tetrahydrocannabinol[Title/Abstract] OR delta(9)-Tetrahydrocannabinol[Title/Abstract] OR Tetrahydrocannabinol[Title/Abstract] OR 9-tetrahydrocannabinol[Title/Abstract] OR delta-9-tetrahydrocannabinol[Title/Abstract] OR tetra-hydrocannabinol*[Title/Abstract] OR sativ* OR indica[Title/Abstract] OR tetra-hydrocannabinol*[Title/Abstract] OR Tetrahydrocannabinol*[Title/Abstract])))

**Muscle relaxant**

((((((((Cyclobenzaprine[MeSH Terms]) OR Cyclobenzaprine[Title/Abstract]) OR Flexeril[Title/Abstract]) OR Amrix[Title/Abstract]) OR Fexmid[Title/Abstract]) OR "FusePaq Tabradol"[Title/Abstract]) OR Lisseril[Title/Abstract])) AND ((((Dronabinol OR Medical Marijuana OR Marijuana Use OR Cannabidiol OR Cannabinoids OR Cannabaceae[MeSH Terms]) OR (Dronabinol [Title/Abstract] OR marinol[Title/Abstract] OR Syndros[Title/Abstract] OR Epidiolex[Title/Abstract] OR Nabilone [Title/Abstract] OR Cesamet[Title/Abstract] OR marijuana[Title/Abstract] OR Sativex[Title/Abstract] OR nabiximol[Title/Abstract] OR marihuana[Title/Abstract] OR Cannabidiol[Title/Abstract] OR cannabi*[Title/Abstract] OR cannabis[Title/Abstract] OR Cannabaceae[Title/Abstract] OR cannabigerolic[Title/Abstract] OR cannabichromene[Title/Abstract] OR Cannabigerol[Title/Abstract] OR delta-9-THC[Title/Abstract] OR Tetrahyrdocannabinol*[Title/Abstract] OR Cannabinoid*[Title/Abstract] OR delta(9)-THC[Title/Abstract] OR 9-ene-Tetrahydrocannabinol[Title/Abstract] OR Δ-9-tetrahydrocannabinol[Title/Abstract] OR delta(9)-Tetrahydrocannabinol[Title/Abstract] OR Tetrahydrocannabinol[Title/Abstract] OR 9-tetrahydrocannabinol[Title/Abstract] OR delta-9-tetrahydrocannabinol[Title/Abstract] OR tetra-hydrocannabinol*[Title/Abstract] OR sativ* OR indica[Title/Abstract] OR tetra-hydrocannabinol*[Title/Abstract] OR Tetrahydrocannabinol*[Title/Abstract])))

**Oncology Medications**

(((Dronabinol OR Medical Marijuana OR Marijuana Use OR Cannabidiol OR Cannabinoids OR Cannabaceae[MeSH Terms]) OR (Dronabinol [Title/Abstract] OR marinol[Title/Abstract] OR Syndros[Title/Abstract] OR Epidiolex[Title/Abstract] OR Nabilone [Title/Abstract] OR Cesamet[Title/Abstract] OR marijuana[Title/Abstract] OR Sativex[Title/Abstract] OR nabiximols[Title/Abstract] OR marihuana[Title/Abstract] OR Cannabidiol[Title/Abstract] OR cannabi*[Title/Abstract] OR cannabis[Title/Abstract] OR Cannabaceae[Title/Abstract] OR cannabigerolic[Title/Abstract] OR cannabichromene[Title/Abstract] OR Cannabigerol[Title/Abstract] OR delta-9-THC[Title/Abstract] OR Tetrahyrdocannabinol*[Title/Abstract] OR Cannabinoid*[Title/Abstract] OR delta(9)-THC[Title/Abstract] OR 9-ene-Tetrahydrocannabinol[Title/Abstract] OR Δ-9-tetrahydrocannabinol[Title/Abstract] OR delta(9)-Tetrahydrocannabinol[Title/Abstract] OR Tetrahydrocannabinol[Title/Abstract] OR 9-tetrahydrocannabinol[Title/Abstract] OR delta-9-tetrahydrocannabinol[Title/Abstract] OR tetra-hydrocannabinol*[Title/Abstract] OR sativ* OR indica[Title/Abstract] OR tetra-hydrocannabinol*[Title/Abstract] OR Tetrahydrocannabinol*[Title/Abstract]))) AND ((((("Busulfan"[Mesh]) OR "Paclitaxel"[Mesh]) OR "temsirolimus" [Supplementary Concept])) OR ((((((((((((((((((((((((((((((((((((busulfan[Title/Abstract]) OR Busulfexm[Title/Abstract]) OR Myleran[Title/Abstract]) OR Mablin[Title/Abstract]) OR Misulban[Title/Abstract]) OR Myeleukon[Title/Abstract]) OR Myelosan[Title/Abstract]) OR Myleran[Title/Abstract]) OR Sulfabutin[Title/Abstract]) OR Busulphan[Title/Abstract]) OR Busilvex[Title/Abstract]) OR Busulfex[Title/Abstract]) OR paclitaxel[Title/Abstract]) OR Taxol[Title/Abstract]) OR Onxol[Title/Abstract]) OR Abraxane[Title/Abstract]) OR Coroflex[Title/Abstract]) OR EndoTAG[Title/Abstract]) OR Genaxol[Title/Abstract]) OR Genetaxyl[Title/Abstract]) OR Intaxel[Title/Abstract]) OR LEP-ETU[Title/Abstract]) OR LipoPac[Title/Abstract]) OR Nab-paclitaxel[Title/Abstract]) OR Nanoxel[Title/Abstract]) OR OncoGel[Title/Abstract]) OR Paclitaxel[Title/Abstract]) OR Taxol[Title/Abstract]) OR Taxus[Title/Abstract]) OR Zisu[Title/Abstract]) OR Anzatax[Title/Abstract]) OR Paxene[Title/Abstract]) OR Praxel[Title/Abstract]) OR temsirolimus[Title/Abstract]) OR rapamycin[Title/Abstract]) OR Torisel[Title/Abstract]))

**Transplant Medications**

(((((((("Cyclosporine"[Mesh]) OR "Everolimus"[Mesh]) OR "Mycophenolic Acid"[Mesh]) OR "Sirolimus"[Mesh]) OR "Tacrolimus"[Mesh])) OR (((((((((((((((((((((((((((((((((((((((((((((((((((Cyclosporin*[Title/Abstract]) OR Neoral[Title/Abstract]) OR CyA-NOF[Title/Abstract]) OR Sandimmune[Title/Abstract]) OR Arpimune[Title/Abstract]) OR Cicloral[Title/Abstract]) OR Consupren[Title/Abstract]) OR Equoral[Title/Abstract]) OR Gengraf[Title/Abstract]) OR Neoplanta[Title/Abstract]) OR Neoral[Title/Abstract]) OR Ramihyphin[Title/Abstract]) OR Restasis[Title/Abstract]) OR Sandimmun*[Title/Abstract]) OR Sangcya[Title/Abstract]) OR CsA-Neoral[Title/Abstract]) OR Restasis[Title/Abstract]) OR SandIMMUNE[Title/Abstract]) OR "Restasis Multidose"[Title/Abstract]) OR Cequa[Title/Abstract]) OR Atopica[Title/Abstract]) OR Everolimus[Title/Abstract]) OR Afinitor[Title/Abstract]) OR Zortress[Title/Abstract]) OR "Afinitor Disperz"[Title/Abstract]) OR Certican[Title/Abstract]) OR Everolimus[Title/Abstract]) OR Xience[Title/Abstract]) OR Zortress[Title/Abstract]) OR Votubia[Title/Abstract]) OR "mycophenolic acid"[Title/Abstract]) OR Cellcept[Title/Abstract]) OR Mycophenolate[Title/Abstract]) OR Myfenax[Title/Abstract]) OR Myfortic[Title/Abstract]) OR sirolimus[Title/Abstract]) OR Rapamune[Title/Abstract]) OR Cypher[Title/Abstract]) OR Sirolimus[Title/Abstract]) OR Supralimus[Title/Abstract]) OR tacrolimus[Title/Abstract]) OR Astagraf[Title/Abstract]) OR Hecoria[Title/Abstract]) OR Prograf[Title/Abstract]) OR Protopic[Title/Abstract]) OR Envarsus[Title/Abstract]) OR Astagraf[Title/Abstract]) OR LCP-Tacro[Title/Abstract]) OR Adoport[Title/Abstract]) OR Capexion[Title/Abstract]) OR Tacni[Title/Abstract]))) AND ((((Dronabinol OR Medical Marijuana OR Marijuana Use OR Cannabidiol OR Cannabinoids OR Cannabaceae[MeSH Terms]) OR (Dronabinol [Title/Abstract] OR marinol[Title/Abstract] OR Syndros[Title/Abstract] OR Epidiolex[Title/Abstract] OR Nabilone[Title/Abstract] OR Cesamet[Title/Abstract] OR marijuana[Title/Abstract] OR Sativex[Title/Abstract] OR nabiximols[Title/Abstract] OR marihuana[Title/Abstract] OR Cannabidiol[Title/Abstract] OR cannabi*[Title/Abstract] OR cannabis[Title/Abstract] cannabis[Title/Abstract] OR Cannabaceae[Title/Abstract] OR cannabigerolic[Title/Abstract] OR cannabichromene[Title/Abstract] OR Cannabigerol[Title/Abstract] OR delta-9-THC[Title/Abstract] OR Tetrahydrocannabinol*[Title/Abstract] OR Cannabinoid*[Title/Abstract] OR delta(9)-THC[Title/Abstract] OR 9-ene-Tetrahydrocannabinol[Title/Abstract] OR Δ-9-tetrahydrocannabinol[Title/Abstract] OR delta(9)-Tetrahydrocannabinol[Title/Abstract] OR Tetrahydrocannabinol[Title/Abstract] OR 9-tetrahydrocannabinol[Title/Abstract] OR delta-9-tetrahydrocannabinol[Title/Abstract] OR tetra-hydrocannabinol*[Title/Abstract] OR sativ* OR indica[Title/Abstract] OR tetra-hydrocannabinol*[Title/Abstract] OR Tetrahydrocannabinol*[Title/Abstract])))

**Vitamin K antagonists (VKAs)**

((((((((((("Acenocoumarol"[Mesh]) OR "clorindione" [Supplementary Concept]) OR "Dicumarol"[Mesh]) OR "diphenadione" [Supplementary Concept]) OR "Ethyl Biscoumacetate"[Mesh]) OR "fluindione" [Supplementary Concept]) OR "Phenprocoumon"[Mesh]) OR "Warfarin"[Mesh])) OR (((((((((((((((((((((((((((((((((((((((((((((((((((((((((Acenocoumarol[Title/Abstract]) OR Acenocouma*[Title/Abstract]) OR Acenocumarol*[Title/Abstract]) OR Acenokumarin[Title/Abstract]) OR Acitrom[Title/Abstract]) OR Ascumar[Title/Abstract]) OR Clorindione[Title/Abstract]) OR Chlophenadione[Title/Abstract]) OR Chlor-athrombon[Title/Abstract]) OR Chlorindionum[Title/Abstract]) OR CI-PID[Title/Abstract]) OR Clorindiona[Title/Abstract]) OR Clorindion[Title/Abstract]) OR Clorindionum[Title/Abstract]) OR Cumachlor[Title/Abstract]) OR Dicoumarol[Title/Abstract]) OR Acadyl[Title/Abstract]) OR Bishydroxycoumarin[Title/Abstract]) OR Cuma[Title/Abstract]) OR Dicoumal[Title/Abstract]) OR Dicoumar*[Title/Abstract]) OR Diphenadione[Title/Abstract]) OR Difenadion[Title/Abstract]) OR Diphacinone[Title/Abstract]) OR Diphénadione[Title/Abstract]) OR Diphenadionum[Title/Abstract]) OR diphenacin[Title/Abstract]) OR difenadion[Title/Abstract]) OR diphacine[Title/Abstract]) OR Dicoumarol[Title/Abstract]) OR Dicumarol[Title/Abstract]) OR biscoumacetate*[Title/Abstract]) OR Ethyldicoumarol[Title/Abstract]) OR Carbethoxydicoumarol[Title/Abstract]) OR Tromexan[Title/Abstract]) OR Pelentan[Title/Abstract]) OR Fluindion*[Title/Abstract]) OR Fluorindione[Title/Abstract]) OR Previscan[Title/Abstract]) OR phenprocoumon[Title/Abstract]) OR Phenylpropyl[Title/Abstract]) OR Phenprocoumalol[Title/Abstract]) OR Phenprocoumarol[Title/Abstract]) OR Phenprogramma[Title/Abstract]) OR Marcoumar[Title/Abstract]) OR Marcumar[Title/Abstract]) OR Falithrom[Title/Abstract]) OR Liquamar[Title/Abstract]) OR warfarin[Title/Abstract]) OR Coumadin[Title/Abstract]) OR Coumarin*[Title/Abstract]) OR Jantoven[Title/Abstract]) OR Coumadine[Title/Abstract]) OR Tedicumar[Title/Abstract]) OR Aldocumar[Title/Abstract]) OR Warfant[Title/Abstract]) OR Marevan[Title/Abstract]))) AND ((((Dronabinol OR Medical Marijuana OR Marijuana Use OR Cannabidiol OR Cannabinoids OR Cannabaceae[MeSH Terms]) OR (Dronabinol [Title/Abstract] OR marinol[Title/Abstract] OR Syndros[Title/Abstract] OR Epidiolex[Title/Abstract] OR Nabilone[Title/Abstract] OR Cesamet[Title/Abstract] OR marijuana[Title/Abstract] OR Sativex[Title/Abstract] OR nabiximols[Title/Abstract] OR marihuana[Title/Abstract] OR Cannabidiol[Title/Abstract] OR cannabi*[Title/Abstract] OR cannabis[Title/Abstract] cannabis[Title/Abstract] OR Cannabaceae[Title/Abstract] OR cannabigerolic[Title/Abstract] OR cannabichromene[Title/Abstract] OR Cannabigerol[Title/Abstract] OR delta-9-THC[Title/Abstract] OR Tetrahydrocannabinol*[Title/Abstract] OR Cannabinoid*[Title/Abstract] OR delta(9)-THC[Title/Abstract] OR 9-ene-Tetrahydrocannabinol[Title/Abstract] OR Δ-9-tetrahydrocannabinol[Title/Abstract] OR delta(9)-Tetrahydrocannabinol[Title/Abstract] OR Tetrahydrocannabinol[Title/Abstract] OR 9-tetrahydrocannabinol[Title/Abstract] OR delta-9-tetrahydrocannabinol[Title/Abstract] OR tetra-hydrocannabinol*[Title/Abstract] OR sativ* OR indica[Title/Abstract] OR tetra-hydrocannabinol*[Title/Abstract] OR Tetrahydrocannabinol*[Title/Abstract])))
